# Systematic Review of the Differential Effects of TGF‐β1 in Ischemic and Hemorrhagic Preclinical Stroke Models

**DOI:** 10.1161/JAHA.124.037890

**Published:** 2025-07-01

**Authors:** Benjamin J. Hewitt, Myah Ali, Jessica Hubbard, Lisa J. Hill, Hannah Botfield

**Affiliations:** ^1^ Biomedical Sciences, School of Infection, Inflammation and Immunology, College of Medicine and Health University of Birmingham Birmingham United Kingdom; ^2^ Inflammation and Ageing, School of Infection, Inflammation and Immunology, College of Medicine and Health University of Birmingham Birmingham United Kingdom

**Keywords:** fibrosis, hemorrhagic stroke, ischemic stroke, transforming growth factor β1, neuro‐inflammation, Growth Factors/Cytokines, Inflammation, Ischemic Stroke, Intracranial Hemorrhage, Cerebrovascular Disease/Stroke

## Abstract

**Background:**

Stroke is a leading cause of death, with patients often experiencing significant disability. Stroke is classified as ischemic, caused by the occlusion of a blood vessel leading to reduction in cerebral blood flow, or hemorrhagic, resulting from the rupture of a vessel that causes bleeding into the brain. Transforming growth factor β1 (TGF‐β1), a pleiotropic cytokine, has been investigated in stroke due to its diverse effects on proliferation, extracellular matrix deposition, and inflammation. This systematic review examined the role of TGF‐β1 in preclinical studies of ischemic and hemorrhagic stroke.

**Methods:**

A search of PubMed, Web of Science, and Scopus databases identified animal studies examining TGF‐β1 signaling as an outcome or intervention. A total of 89 studies were included: 68 on ischemic stroke and 21 on hemorrhagic stroke. Studies were assessed for bias following the SYRCLE (Systematic Review Centre for Laboratory Animal Experimentation) guidelines, followed by extraction of methodology and the role of TGF‐β1.

**Results:**

Compliance with SYRCLE guidelines was found to be low, and the methodological approaches to stroke models were variable. A range of interventions were shown to modify TGF‐β1 expression or signaling, with exogenous TGF‐β1 improving outcomes in all ischemic stroke studies. TGF‐β1 was found to play a protective role in 76% of ischemic stroke studies but was only protective in 33% of hemorrhagic stroke studies, with likely involvement in fibrosis development in the latter.

**Conclusions:**

Our findings suggest a marked difference in TGF‐β1 function between these types of stroke, and it is hypothesized that blood cytotoxicity following hemorrhagic stroke may generate more sustained TGF‐β1 expression than that seen in ischemic stroke. This may lead to TGF‐β1–mediated fibrosis and hydrocephalus, as opposed to the neuroprotective role played by the same molecule following ischemic stroke. These findings highlight the possible clinical utility of exogenous TGF‐β1 therapies after ischemic stroke, and TGF‐β1 inhibitors after hemorrhagic stroke, to reduce morbidity and disability caused by these events.

## Graphical abstract

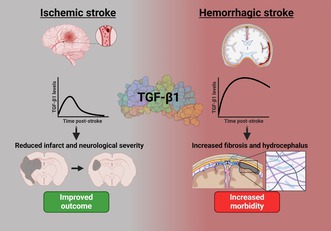

**Nonstandard Abbreviations and Acronyms**
BBB: blood–brain barrier
CBF: cerebral blood flow
GMH: germinal matrix hemorrhage
ICH: intracerebral hemorrhage
IVH: intraventricular hemorrhage
LRG1: leucine‐rich α‐2 glycoprotein 1
MCAO: middle cerebral artery occlusion
NSS: Neurological Severity Score
RoB: Risk of Bias
SAS: subarachnoid spave
SYRCLE: Systematic Review Centre for Laboratory Animal Experimentation
TGF‐β1: transforming growth factor β1
ZEB1: zinc finger E‐box binding homeobox 1

**Research Perspective**

**What Is New?**

- Systematic analysis of preclinical models of ischemic and hemorrhagic stroke showed that transforming growth factor β1 correlated with or caused a protective effect in 76% of ischemic stroke studies but only 33% of hemorrhagic stroke studies.
- Injection of exogenous transforming growth factor β1 was shown to improve neurological scoring, recovery, or infarct size in all included ischemic stroke studies that examined these as end points.

**What Question Should Be Addressed Next?**

- Is there a clinical potential for the use of exogenous transforming growth factor β1 following ischemic stroke, and for transforming growth factor β1 blockers or antagonists in patients with hemorrhagic stroke to prevent posthemorrhagic hydrocephalus or fibrosis?

Stroke remains a leading cause of death in the Western World,^1^ with an incidence of ≈3% in the general population^2^ and a case fatality rate of ≈46% and 61% for ischemic stroke and intracranial hemorrhage, respectively.^3^ In addition, survivors frequently experience significant morbidity, with up to 50% becoming chronically disabled.^4^ With this high rate of mortality and morbidity, stroke poses a significant economic burden, consuming up to 1.7% of total health expenditure in Europe, in addition to costs incurred by social care demands and productivity loss.^5^ This public health burden is expected to grow due to an increasingly aging population coupled with a continual rise in risk factors including hypertension, poor diet, and abdominal obesity.^6^

Stroke is broadly separated into ischemic and hemorrhagic types, where both result in a reduction in blood and oxygen delivery to the brain tissue, rapidly causing a loss in cell function and viability.^7^ Ischemic stroke is the more prevalent form, accounting for ≈80% of all stroke cases,^4^ and is typically triggered by embolic occlusion of blood flow to the brain.

Hemorrhagic strokes are less common, accounting for ≈20% of stroke cases,^4^ though with markedly greater mortality risk than ischemic stroke.^8^ Hemorrhagic stroke is marked by blood vessel rupture, causing bleeding into the parenchyma (intracerebral hemorrhage [ICH]), ventricular system (geminal matrix hemorrhage [GMH]/intraventricular hemorrhage [IVH]), or subarachnoid space (subarachnoid hemorrhage [SAH]) with a resultant reduction in tissue oxygenation.^9^ While these conditions are all linked by bleeding into tissue or cerebrospinal fluid (CSF)–filled spaces, the precipitating event and location of the bleed may cause marked variations in outcome between the different forms of hemorrhagic stroke. As a whole, hemorrhagic strokes generate free blood within the dural spaces, parenchyma, or ventricles, resulting in hematoma formation and cerebral swelling. As in ischemic stroke, early diagnosis and treatment are crucial to provide the patient with the greatest chance of survival without significant neurological deficit.

Inflammation plays a key role in both ischemic and hemorrhagic stroke, triggered either by tissue damage or the response of brain tissue to free blood.^10^ The inflammatory cascade comprises a range of receptors, cells, and cytokines, although there is a growing focus on the pleiotropic cytokine transforming growth factor β1 (TGF‐β1) as a key element in determining morbidity in both ischemic and hemorrhagic stroke. TGF‐β1 has been shown to exert influence over proliferation, differentiation, apoptosis, and immune cell activation^11^ in a range of cell types via the Smad signaling pathway.^12^ TGF‐β1 has also been shown to upregulate astrocyte type 1 plasminogen activator inhibitor,^13^ increase angiogenesis,^14^, ^15^ and reduce inflammation by downregulating nuclear factor κβ.^16^ In addition, TGF‐β1 activation plays a key role in extracellular matrix remodeling, controlling the deposition of extracellular matrix proteins including fibronectin and collagen^17^ via Smad2/3 signaling.^18^ While the TGF‐β1 pathway is typically tightly controlled, severe trauma—such as that generated by stroke—may lead to dysregulation, causing excessive and disordered extracellular matrix deposition, known as fibrosis.^19^

This systematic review aims to investigate the role of TGF‐β1 in preclinical animal models of ischemic and hemorrhagic stroke. Little research has been published comparing the roles of TGF‐β1 in these types of stroke, with most human studies limited to investigation of TGF‐β1 levels in CSF. However, preclinical animal models enable investigation into not only TGF‐β1 expression but also signaling pathways, fibrosis formation, tissue viability, and other relevant end points not obtainable in human studies. Improved understanding of the influence of TGF‐β1 upregulation in stroke may aid in the development of new therapies to improve the prognosis of this serious, life‐threatening condition.

## METHODS

### Protocol Registration

The research project protocol for this systematic review has been registered on PROSPERO, with reference number CRD42022341574. PRISMA (Preferred Reporting Items for Systematic Reviews and Meta‐Analyses) guidelines^20^ were followed throughout this review (Table S1).

### Search Strategy and Data Sources

A literature search was performed (last search January 18, 2024, for all) on PubMed for “((SAH) OR (subarachnoid haemorrhag*) OR (subarachnoid hemorrhag*) OR (ischem* stroke) OR (ischaem* stroke) OR (IVH) OR (intraventricular haemorrhag*) OR (intraventricular hemorrhag*) OR (ICH) OR (intracerebral haemorrhag*) OR (intracerebral hemorrhag*) OR (intracranial haemorrhag*) OR (intracranial hemorrhag*)) AND ((transforming growth factor beta 1) OR (TGFβeta1))”.

Web of Science search terms were “ALL=(((SAH) OR (subarachnoid h$emorrhag*) OR (isch$em* stroke) OR (IVH) OR (intraventricular h$emorrhag*) OR (ICH) OR (intracerebral h$emorrhag*) OR (intracranial h$emorrhag*)) AND ((transforming growth factor beta 1) OR (TGFβeta1)))”.

Scopus search terms were “TITLE‐ABS‐KEY (((sah) OR (subarachnoid AND haemorrhag*) OR (subarachnoid AND hemorrhag*) OR (ischem* AND stroke) OR (ischaem* AND stroke) OR (ivh) OR (intraventricular AND haemorrhag*) OR (intraventricular AND hemorrhag*) OR (ich) OR (intracerebral AND haemorrhag*) OR (intracerebral AND hemorrhag*) OR (intracranial AND haemorrhag*) OR (intracranial AND hemorrhag*)) AND ((transforming AND growth AND factor AND beta 1) OR (TGFβeta1)))”.

### Eligibility Criteria

#### Screening Process, Data Extraction, and Quality Assessment

Screening of titles and abstracts was performed by 3 independent investigators (BJH, MA, HB) using the systematic review management tool Covidence.^21^ All selections were made independently and blinded to the other investigator's choice, and any conflicts were resolved by consensus or consultation with another investigator (LJH) if required. Duplicate, irrelevant, and unavailable articles were excluded at this stage. Reviews and non‐English articles were also excluded. Full text articles were then read and selected based on the defined eligibility criteria, following the same process. Articles were designated as “full text unavailable” if the full text was not freely available via a search engine, via journal websites with access provided by University of Birmingham Library Services, or on publicly accessible repositories (ie, ResearchGate).

Primary data extraction and quality assessment were performed by one investigator (BH) using Covidence, followed by checking and comparison to the full text by a second investigator (HB). Any conflicts were resolved as previously discussed. Quality assessment of each article followed the best practices for bias assessment in preclinical animal studies defined by SYRCLE's Risk of Bias (RoB) tool.^22^ Any of the SYRCLE RoB signaling questions that were not explicitly discussed within the work were recorded as a “no” response. RoB was then quantified by adding together the number of signaling questions answered to a satisfactory standard. This generated a score from 0 to 10, where 0 was the highest RoB and 10 was the lowest.

#### Population and Model

All in vitro and human in vivo studies were excluded during screening. Accepted models of hemorrhagic stroke included injection of blood/blood products, collagenase injections, endovascular perforation, and any relevant genetic models. Ischemic stroke models included both transient and permanent middle cerebral artery occlusion (MCAO), with hypoxia‐induced global ischemia models excluded.

#### Intervention and Comparators

Any intervention, including application of TGF‐β1, was included. No comparator group was required for inclusion, and no exclusion criteria were set for interventions or comparators.

### Outcomes

Accepted outcome measures included any alteration in TGF‐β1 or the TGF‐β1 signaling pathway. However, studies in which TGF‐β1 was measured as part of a large panel of inflammatory molecules (eg, RNA sequencing) where TGF‐β1 signaling was not one of the primary outcomes were excluded.

### Data Collection, Synthesis, and Presentation

Included papers were read to extract data from text, figures, and supplemental data if required. Information on methods, including the type of stroke modeled, induction techniques, interventions, animal species, and the number of animals, was extracted as part of the study characteristics. Measured outcomes and the impact of any interventions was recorded, in addition to the general conclusion formed by the data, with particular focus on TGF‐β1–dependent outcomes or changes in TGF‐β1 signaling. The role of TGF‐β1 was then summarized briefly and classified as being protective or harmful based on available data within the paper. “Protective” and “harmful” were defined as causing an objective reduction or increase, respectively, in mortality, morbidity, or relevant molecular markers. “Unclear” was assigned to studies showing mixed outcomes, or studies in which the role of TGF‐β1 was not clearly described. Data extraction and outcome classifications were performed by one investigator (BJH) and checked by a second (HB), with any conflicts discussed and resolved with a third investigator (LJH) if required. A PRISMA flowchart was then generated, in addition to a narrative synthesis of the data. Figures were generated using GraphPad Prism 10.0 (GraphPad Software, LLC).

Where required, TGF‐β1 levels were interpreted from graphs and figures using the tool WebPlotDigitizer,^23^ with all plots interpreted by the same user to minimize interoperator variability.

### Statistical Analysis

A meta‐analysis was performed for ischemic stroke studies introducing exogenous TGF‐β1 and for hemorrhagic studies of TGF‐β1 inhibitors or antagonists, with forest plots generated using RevMan (The Cochrane Collaboration).^24^ Standardized mean difference was employed as the effect size measure, and both random‐effects and fixed‐effects analysis to assess sensitivity were performed. Infarct size was used as an outcome measure in ischemic stroke studies, while lateral ventricular size was used for hemorrhagic studies. Values were either taken directly from articles or extracted using WebPlotDigitizer as above. Subgroup analysis was not performed due to high diversity in the model, species, treatment, and treatment route providing no two studies had the same characteristics.

## RESULTS

### Search Results

**Figure 1:**
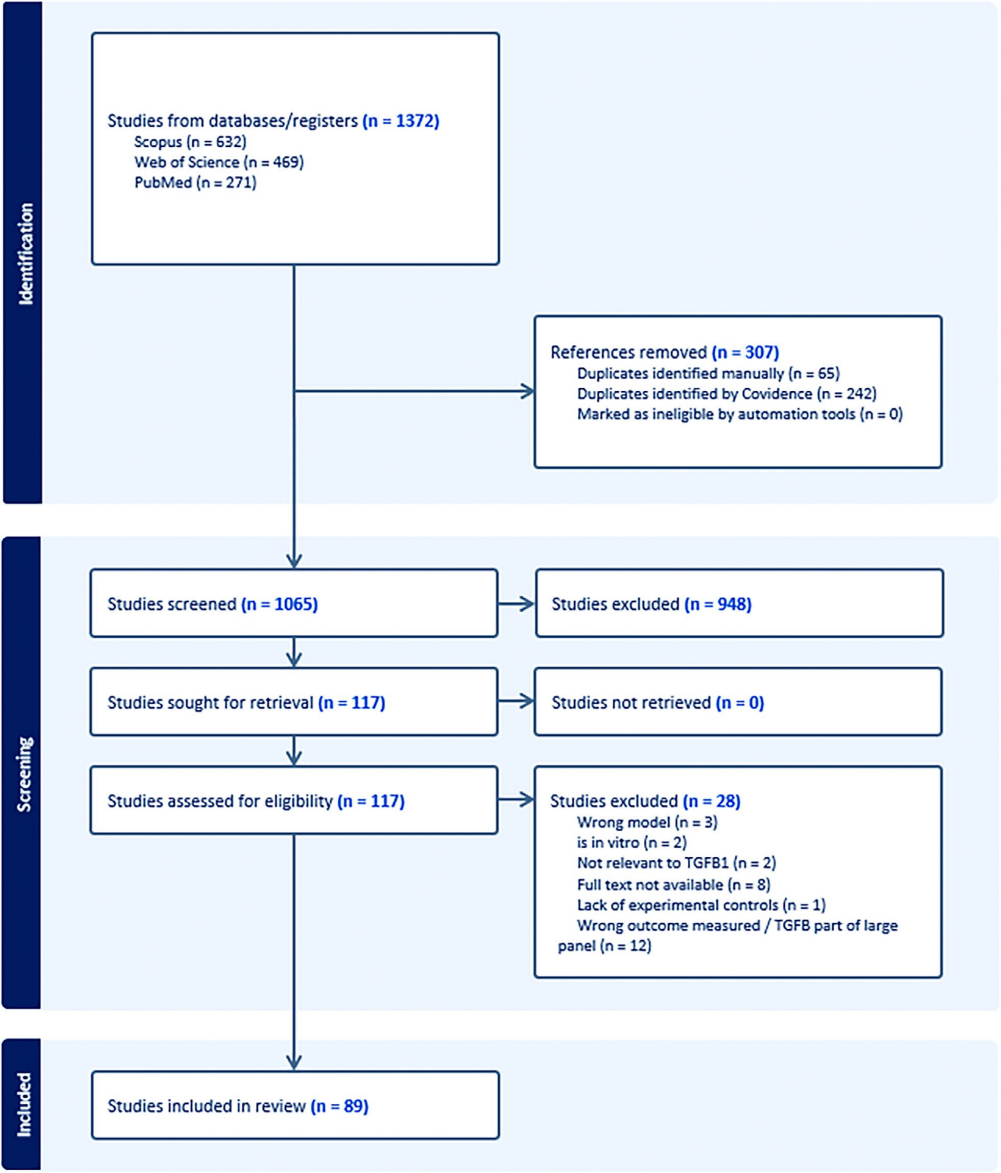
PRISMA flow chart of the study screening and selection protocol. PRISMA indicates Preferred Reporting Items for Systematic Reviews and Meta‐Analyses; TGF‐β, transforming growth factor β; and TGF‐β1, transforming growth factor β1.

A total of 1372 articles were collected using Scopus (n=632), Web of Science (n=469), and PubMed (n=271), with 307 duplicates removed. Following title and abstract screening, 948 irrelevant articles were removed. A total of 117 full‐text articles were assessed for inclusion, with 28 excluded (Figure 1). Three of these were excluded due to use of an inappropriate model,^25^, ^26^, ^27^ 2 for being performed in vitro,^28^, ^29^ 2 due to not being relevant to TGF‐β1,^30^, ^31^ 8 for not having full text available,^32^, ^33^, ^34^, ^35^, ^36^, ^37^, ^38^, ^39^ 1 due to a lack of experimental controls,^40^ and 12 for measuring inappropriate outcomes or measuring TGF‐β1 as part of a large panel of molecules.^41^, ^42^, ^43^, ^44^, ^45^, ^46^, ^47^, ^48^, ^49^, ^50^, ^51^, ^52^ As such, 89 studies were included for analysis in the present review. Full characteristics of these studies are included in the (Tables S2 and S3).

### Modeling of Stroke in Preclinical Animal Studies: Study Design

#### Ischemic Stroke Models

Some studies reviewed used multiple methods or animal species within the same body of work, which are counted separately below. Of the 89 included studies, 68 modeled ischemic stroke, with MCAO by insertion of an intraluminal filament being the most widely used model. Of these, 52 studies employed the transient MCAO or MCAO/reperfusion model, while 12 utilized a permanent MCAO approach, typically with clamps or cauterization. A further 4 studies used photothrombosis to induce focal cortical ischemia following administration of a photosensitizing agent, while 2 studies utilized the injection of autologous blood clots to the internal carotid artery.

**Figure 2:**
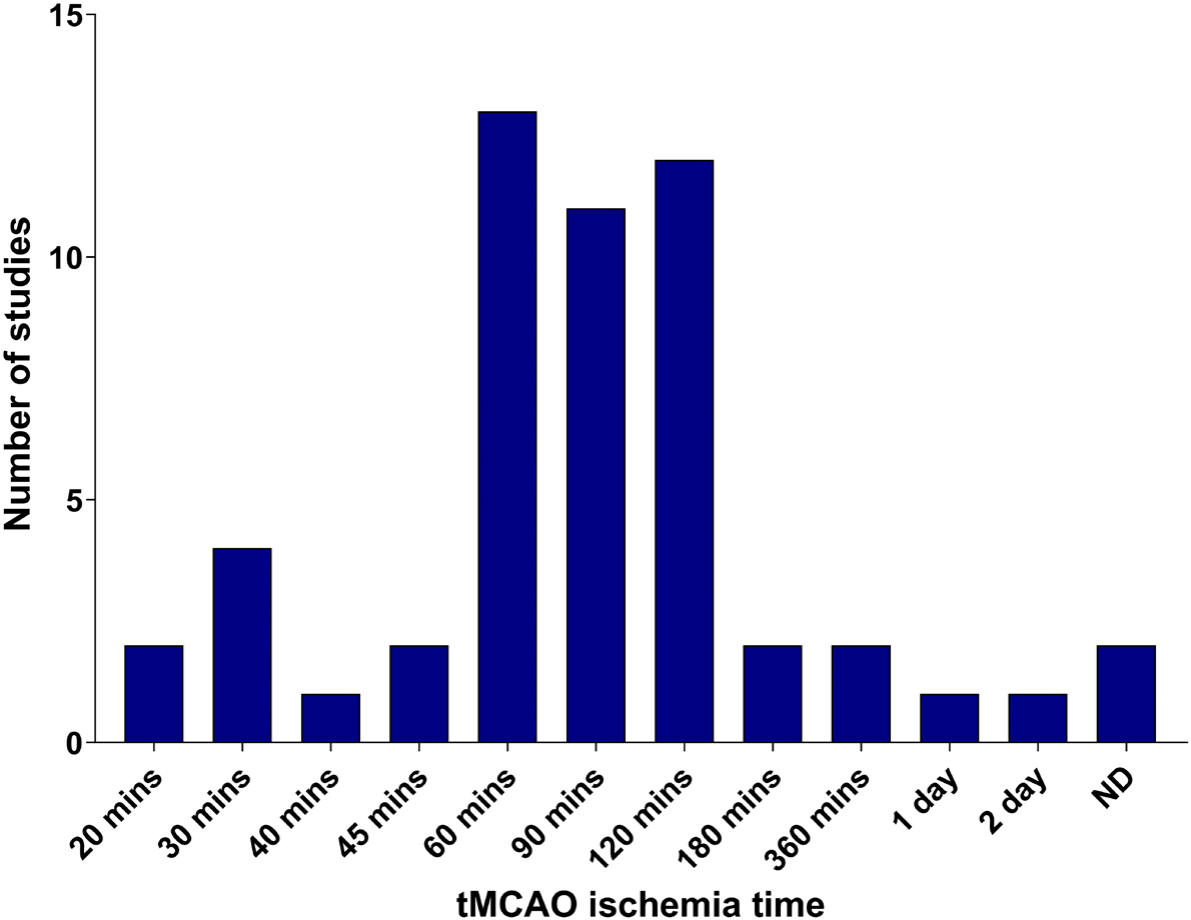
Histogram of transient MCAO ischemia time in ischemic stroke studies. MCAO indicates middle cerebral artery occlusion; and ND, not disclosed.

MCAO time was highly variable within transient MCAO studies, with a range between 20 minutes to 2 days of occlusion before reperfusion, as is depicted in Figure 2. The most common occlusion times were 60, 90, and 120 minutes, collectively accounting for 36 of the 89 included studies.

#### Hemorrhagic Stroke Models

Of the 89 included studies, 21 modeled hemorrhagic stroke; 8 SAH, 7 ICH, and 6 GMH/IVH. SAH was induced by endovascular perforation of the middle cerebral artery or intracranial internal carotid artery, or by injection of blood/blood products into the cisterna magna. ICH was induced by injection of blood products or collagenase into the brain parenchyma, and IVH/GMH was induced by intracerebroventricular injection of blood, collagenase, or glycerol.

#### Animal Species and Strains Used

**Figure 3:**
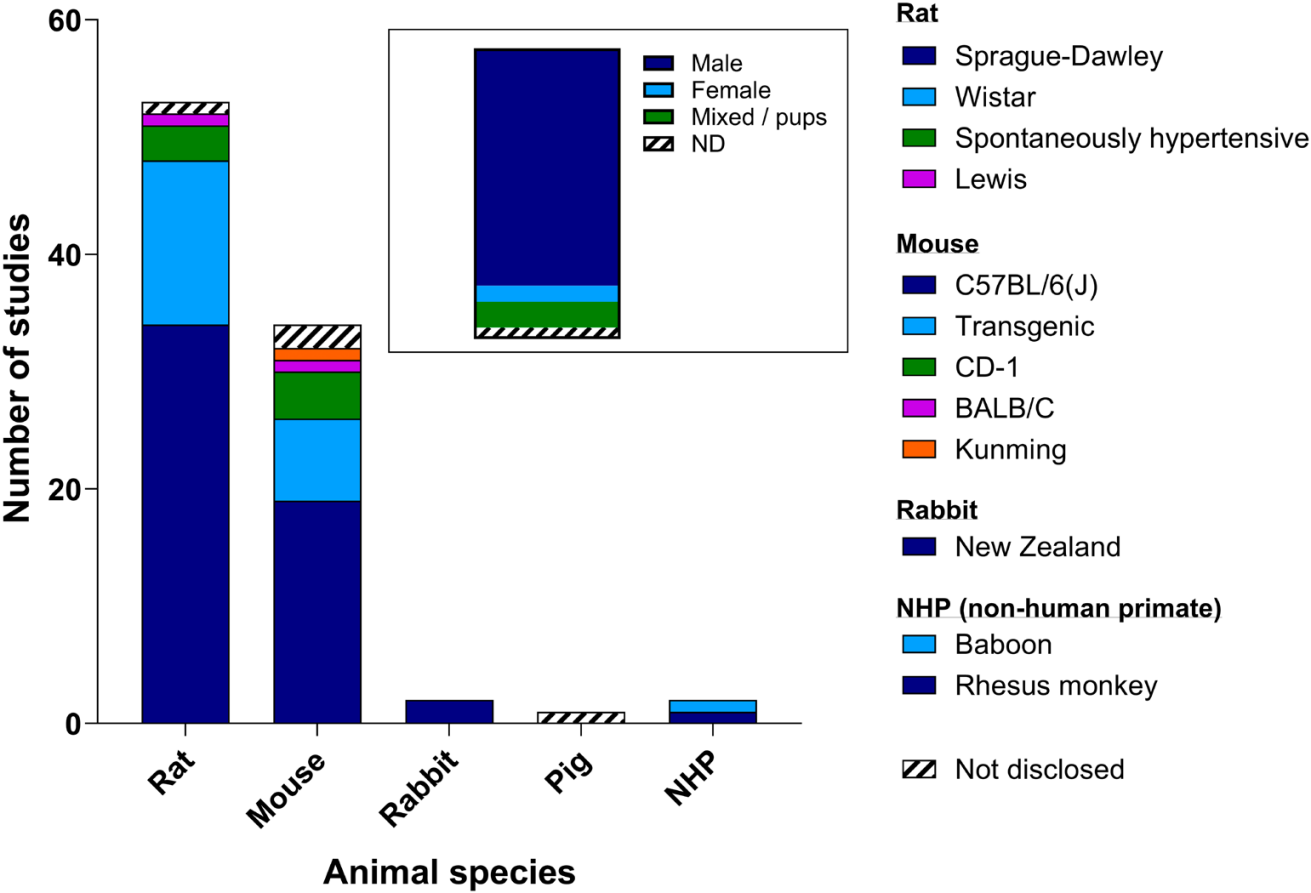
The distribution of different animal species within the included studies, broken down into strain. Inset shows the distribution of animal sexes used within the same set of studies. ND indicates not disclosed; and NHP, nonhuman primate.

Rodent models were used in nearly all included studies for both forms of stroke (Figure 3). Of the 89 included studies, 53 used rats, with 33 studies using Sprague–Dawley rats, 14 using Wistar rats, 3 using spontaneously hypertensive rats, 1 using Lewis rats, and 1 that did not disclose the rat species used. Of the 31 studies that used mice, 19 used the C57BL/6 or C57Bl/6J strains, 4 used the CD‐1 strain, 1 used Kunming mice, and 1 used the BALB/C strain. Seven studies used transgenic mice, and 2 studies did not disclose the mouse strain used. In addition, 2 studies used white New Zealand rabbits and 1 used piglets. Only 2 studies utilized nonhuman primates, with 1 using rhesus monkeys and 1 using baboons.

Male animals were used in 73 of the included 89 studies (81.8%), with only 8 studies using mixed sex populations and 5 using only female animals (Figure 3, inset). In addition, 3 studies failed to disclose the sex of the animals employed within the study.

#### TGF‐β1 Measurements

TGF‐β1 was measured directly in 52 ischemic stroke studies (76%) and 13 hemorrhagic stroke studies (62%). In ischemic stroke, the main measures were semiquantitative, employing polymerase chain reaction (16 studies) and in situ hybridization (4 studies) to assess gene expression, and Western blot (26 studies) and immunohistochemistry (20 studies) to assess protein in a variety of samples, including whole brain, contralateral hemisphere, ipsilateral hemisphere, penumbra, core, and peri‐infarct cortex. Assessments were usually performed within the first few days of stroke induction, with only a few studies conducting time course experiments. One study^53^ demonstrated that TGF‐β1 gene expression peaked at 7 days after MCAO in the core, showed a slight increase in the peri‐infarct area, but remained low in the contralateral hemisphere, showing different patterns of expression in the different areas of the lesion. Only 6 studies used enzyme‐linked immunosorbent assay or Luminex to quantify TGF‐β1 levels, mainly demonstrating that TGF‐β1 increased in brain tissue following ischemic stroke, although the values were highly variable (186.2 to 53 200 pg/g in MCAO, as shown in Table S4).

In hemorrhagic stroke, Western blot (7 studies) and enzyme‐linked immunosorbent assay (6 studies) were the most frequent techniques for measuring TGF‐β1, followed by polymerase chain reaction (4 studies) and immunohistochemistry (3 studies). TGF‐β1 was mainly measured by enzyme‐linked immunosorbent assay in CSF from IVH or SAH studies and demonstrated an increase in the levels of TGF‐β1 protein over 2 to 3 weeks,^54^, ^55^, ^56^, ^57^ with consistent concentrations (maximum of 69.3–315.5 pg/mL). One study in rabbits demonstrated a reduction in CSF TGF‐β1 following IVH, but their control levels were variable.^58^

#### Protective or Harmful Role of TGF‐β1

**Figure 4:**
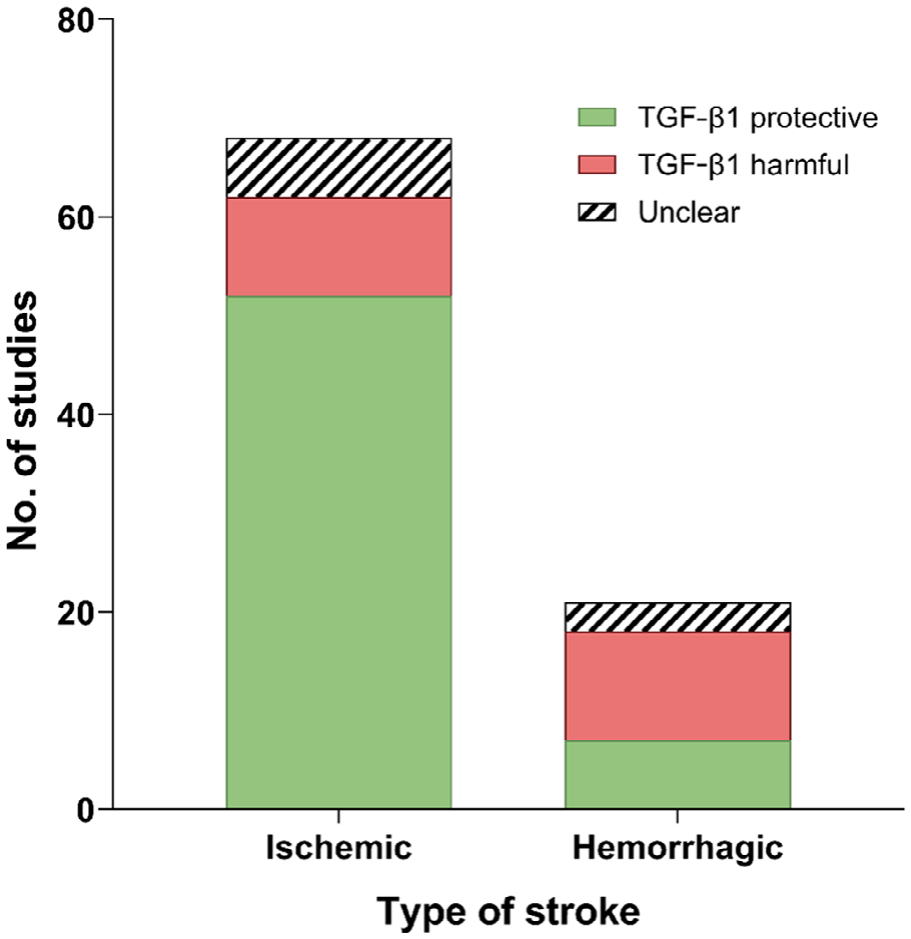
The role of TGF‐β1 in preclinical studies of ischemic and hemorrhagic stroke. TGF‐β1 indicates transforming growth factor β1.

Preclinical models of ischemic and hemorrhagic stroke showed a split in the possible protective role of TGF‐β1, as depicted in Figure 4. Fifty‐two of the included 68 ischemic stroke studies (76%) showed a protective effect of TGF‐β1 supplementation or expression, with 10 (15%) showing a harmful effect and 6 (9%) showing an unclear effect. Within hemorrhagic stroke studies, 7 of the 21 included studies (33%) showed a protective effect of TGF‐β1, 11 (57%) showed a harmful effect, and 3 were unclear (14%).

#### TGF‐β1 in Preclinical Ischemic Stroke Studies

Table 1, ^14^, ^15^, ^16^, ^53^, ^59^, ^60^, ^61^, ^62^, ^63^, ^64^, ^65^, ^66^, ^67^, ^68^, ^69^, ^70^, ^71^, ^72^, ^73^, ^74^, ^75^, ^76^, ^77^, ^78^, ^79^, ^80^, ^81^, ^82^, ^83^, ^84^, ^85^, ^86^, ^87^, ^88^, ^89^, ^90^, ^91^, ^92^, ^93^, ^94^, ^95^, ^96^, ^97^, ^98^, ^99^, ^100^, ^101^, ^102^, ^103^, ^104^, ^105^, ^106^, ^107^, ^108^, ^109^, ^110^, ^111^, ^112^, ^113^, ^114^, ^115^, ^116^, ^117^, ^118^, ^119^, ^120^, ^121^, ^122^ details the model, role of TGF‐β1, study type, and outcome for each included ischemic stroke study.

**Table 1:** Ischemic Stroke Study Characteristics

Reference #	Model	Role of TGF‐β1	Study type and relevant interventions	TGF‐β1 outcome
59	Male Wistar rats, 2‐h tMCAO	TGF‐β1 was increased following ischemic stroke Propolis increased TGF‐β1 levels further, suggesting a protective role	Interventional	Protective
60	Male C57BL/6 mice, tMCAO (time ND)	Activation of TGF‐β1 by ZEB1 reduces infarct volume and maintains BBB	Interventional, including TGF‐β1 knockdown	Protective
61	Male SHR rats, 90‐min tMCAO	TGF‐β1 increased by minocycline treatment, correlating with improved outcomes	Interventional	Protective
62	Male SD rats, pMCAO via embolus injection	TGF‐β1 increase correlated with reduction in NSS and increase in vascular density	Interventional	Protective
63	Male SD rats, 2‐h tMCAO	Upregulation of TGF‐β1 pathway leads to reduced apoptosis markers after MCAO	Interventional, including Smad3 overexpression	Protective
64	Male BALB/c mice, 20‐min tMCAO	Reduced TGF‐β1 activation by suppressing RMST with miR‐221‐3p resulted in better outcomes	Interventional	Harmful
65	Male SD rats, 2‐h tMCAO	Increased in expression by MCAO, but also significantly increased by treatment with UK, correlating with reduced infarct volume	Interventional	Protective
66	Male SD rats, 90‐min tMCAO	Inhibiting TGF‐β1 reduced markers of isoflurane postconditioning, which had been shown to reduce NSS, infarct volume, and neuronal apoptosis	Interventional, TGF‐β antagonist	Protective
67	Male C57BL/6 mice, pMCAO via cauterization	TGF‐β1 worsens fibrosis and neurological recovery and modifies perivascular CSF flow in aged mice MCAO model	Interventional, TGF‐β antagonist and exogenous TGF‐β1	Harmful
68	Male SD rats, 2‐h tMCAO	Suppression of TGF‐β1/SMAD3 by miR‐323 reduced neurotoxicity in cerebral infarct model	Observational	Protective
69	Male SD rats. 90‐min tMCAO	Increased TGF‐β1 expression following isoflurane postconditioning correlates with reduced NSS, reduced infarct volume, and increased viable neuron density	Interventional, including TGF‐β1 inhibitor	Protective
70	Male SD rats, tMCAO for 6 h, 1 d, and 2 d	TGF‐β1 slightly upregulated by NBP, correlated with improved NSS and infarct size	Interventional	Protective
71	Male Lewis rats, 3‐h tMCAO	Increased secretion of TGF‐β1 correlates with reduced infarct size	Interventional	Protective
53	Male Wistar rats, 90‐min tMCAO	Increase in TGF‐β1 poststroke may increase number of NG2+ microglia, which may improve outcome	Observational	Protective
72	Male C57BL/6 mice, both wild‐type and transgenic: (CD45.1)(B6.SJL‐PtprcaPepcb/BoyJ), (CCR2−/−)(B6.129S4‐Ccr2tm1Ifc/J) and (CX3CR1−/− (B6.129P‐Cx3cr1tm1Litt/J)) 40‐min tMCAO or cortical photothrombosis	TGF‐β1 reduced HT in monocytes/macrophages‐depleted brains and corrected neovessel morphology	Interventional, exogenous TGF‐β1	Protective
73	Male SD rats, 90‐min tMCAO	Increased TGF‐β1 expression and Smad3 activation correlated with reduced infarct size	Interventional, exogenous TGF‐β1 and TGF‐β1 inhibitor	Protective
74	Male albino Wistar rats, 20‐min tMCAO	TGF‐β1 supplementation reduced infarct size post‐MCAO	Interventional, exogenous TGF‐β1 and TGF‐β1 inhibitor	Protective
75	Male mice (species ND), 45‐min tMCAO or combined cortical photothrombosis and proximal MCA ligation	Increased TGF‐β1 levels reduced HT in ischemic stroke models	Interventional	Protective
76	Male SD rats, 2‐h tMCAO	Increase in TGF‐β1 protein and p‐Smad2/3 correlated with improved outcomes	Interventional, including TGF‐β1 knockdown	Protective
77	Male SHR rats, 1‐h tMCAO	Suggested neuroprotective effect when taking primary data with other literature in discussion	Observational	Protective
78	Male SD rats, pMCAO	Decreased TGF‐β1 and Smad2/3 expression reduced proinflammatory signaling and improved outcomes	Interventional	Harmful
79	Male CD‐1 mice, 30‐min tMCAO	TGF‐β1 overexpression reduced infarct volume and increased MIP and MCP expression	Interventional, TGF‐β1 knockdown	Protective
14	Male SD rats, pMCAO	TGF‐β1 protein increased post‐MCAO, correlating with increased neovascularization	Observational	Protective
80	Male Wistar rats, 1‐h tMCAO	TGF‐β1 increased post‐MCAO, especially in cortex	Observational	Unclear
81	Male SD rats, autologous clot injection into internal carotid artery	TGF‐β1 protects against HT induced by rt‐PA treatment post‐thromboembolic MCAO	Interventional, including exogenous TGF‐β1	Protective
82	Male SHR rats, pMCAO	TGF‐β1 increased in the subacute stage of stroke, suggesting a link to healing processes	Observational	Protective
83	Male Wistar rats, systemic hypotension combined with bilateral carotid artery occlusion, 10‐min ischemic time	TGF‐β1 reduces injury to hippocampal neurons and stabilizes neuron Ca^2+^ homeostasis	Interventional, exogenous TGF‐β1	Protective
84	Male SD rats, 90‐min tMCAO	TGF‐β1 injection reduced infarct volume and motor deficit following MCAO	Interventional, media containing TGF‐β1 protein	Protective
85	Male C57BL/6J mice, 30‐min tMCAO	TGF‐β1 increased vascular remodeling and tissue repair	Observational	Protective
16	Male Wistar rats, 90‐min tMCAO	TGF‐β1 increased expression in ischemic core at 7 DPI by tMCAO	Interventional, exogenous TGF‐β1	Unclear
86	Male C57BL/6 mice, 90‐min tMCAO	Intranasal TGF‐β1 reduces infarct volume, improves neurofunction, and decreases apoptosis	Interventional, exogenous TGF‐β1	Protective
87	Male Wistar rats, either 1‐h tMCAO or pMCAO	Induced rapidly post‐tMCAO, even in regions that do not express TGF‐β1 when uninjured	Observational	Unclear
88	Male SD rats, 30‐min tMCAO	Antagonism of TGF‐β1 increases size of excitotoxicity‐ and MCAO‐induced lesions	Interventional, including exogenous TGF‐β1 and antagonist	Protective
89	Male C57BL/6J mice, pMCAO	TGF‐β1 expression downregulated post‐MCAO by MSC treatment	Interventional	Unclear
90	Male and female New Zealand white rabbits, autologous clot delivery to internal carotid artery	Reduced infarct size after 4 h of ischemia and increased CBF after 10, 60, and 120 min	Interventional, including exogenous TGF‐β1	Protective
91	Male CD‐1 mice, 30‐min tMCAO	TGF‐β1 may suppress proapoptotic protein Bad, correlating with improved outcomes	Interventional, including TGF‐β1 overexpression	Protective
92	Male SD rats, 2‐h tMCAO	TGF‐β1 activation by IL‐4 reduces infarct volume, apoptosis, and NSS	Interventional	Protective
93	Male C57BL/6 mice, 1‐h tMCAO	TGF‐β1/Smad2/3 pathway upregulation reduced neurological deficits post‐MCAO	Interventional	Protective
94	Male baboons, pMCAO	TGF‐β1 expression increased following ischemic stroke	Observational	Unclear
95	Male SD rats, 1‐h tMCAO	TGF‐β1 upregulated by laser treatment, correlating with reduced NOS expression	Interventional	Protective
96	Female transgenic (Ast‐Tbr2DN) mice, cortical photothrombosis	TGF‐β1 reduces inflammation in peri‐infarct cortex, reduces infarct size, and improves motor outcomes after stroke	Interventional, genetic reduction of TGF‐β1 signaling	Protective
97	Male Wistar rats, pMCAO by autologous clot delivery to MCA	TGF‐β1 pathway upregulation was correlated with spontaneous recovery from MCAO	Observational	Protective
98	Male SD rats, 2‐h tMCAO	TGF‐β1 knockdown correlated with worsened infarct volume and NSS/functional recovery	Interventional	Protective
99	Male mice (species ND), 90‐min tMCAO	Increased serum TGF‐β1 levels by hUS‐MSCs suggested to be neuroprotective	Interventional	Protective
100	Male Wistar rats, either 1‐h tMCAO or pMCAO	TGF‐β1 receptor expression is induced after tMCAO and may contribute to recovery	Observational	Protective
101	Male Wistar rats, pMCAO	Suppression of TGF‐β1 may improve neurological outcome in embolic stroke model	Interventional	Harmful
102	Male SD rats, 2 h‐tMCAO	TGF‐β1 pathway upregulation correlated with reduced infarct volume and improved NSS 28 days after MCAO	Interventional	Protective
103	Unsexed rat pups and transgenic (Cx3cr1(GFP+) /Ccr2(RFP+)) mice	TGF‐β1 prevents HT in neonatal MCAO model	Interventional, including exogenous TGF‐β1	Protective
104	Male SD rats, 2‐h tMCAO	Reduction in TGF‐β1 levels (and ALK5 levels) led to reduction in ROS damage and thus reduced the effects of tMCAO	Interventional, including ALK5 inhibitor	Harmful
105	Male SD rats, 90‐min tMCAO	TGF‐β1 correlated with reduction in neurological deficits and infarct volume	Interventional including TGF‐β1 antagonist	Protective
106	Male SD rats, pMCAO	Upregulation of TGF‐β1 by TSP4‐overexpressing BMSCs improved neurological function	Interventional	Protective
107	Male C57BL/6 mice, 1‐h tMCAO	TGF‐β1 upregulated by overexpression of LRG1, promoting apoptosis and increasing infarct size and neurological deficit post‐tMCAO	Interventional	Harmful
108	Rhesus monkeys, 82‐ to 162‐min tMCAO	TGF‐β1 localized with CD68+ microglia/macrophages in the subacute phase (indicating healing response)	Observational	Protective
109	Female C57BL/6J mice, cortical photo thrombosis	TGF‐β1 upregulated postischemic stroke	Observational	Unclear
110	Male transgenic mice (Rosa26mTmG/+ crossed with dh5‐cre /ERT2‐IRES‐tdTomato), 1‐h tMCAO	Increased TGF‐βRI increased vascular fibrosis post‐MCAO	Interventional, including TGF‐βRI antagonist	Harmful
111	Male C57BL/6 mice, 6‐h tMCAO	Decreased expression of Smad2/3 pathway correlated with improved outcome post‐pMCAO	Interventional	Harmful
112	Male SD rats, 2‐h tMCAO	Overexpression of ALK5 correlated with improved outcomes	Interventional, including ALK5 overexpression	Protective
113	Male C57BL/6 and B3galt2 knockout mice, 90‐min tMCAO	Treatment with TGF‐β1 improved neurological outcomes post‐MCAO	Interventional, including exogenous TGF‐β1	Protective
114	Male SD rats, 90‐min tMCAO	TGF‐β1 upregulation by 1,25‐D3 produced a neuroprotective effect	Interventional	Protective
115	SD rats (sex ND), 1‐h tMCAO	Reduction in TGF‐β1 correlated with improved outcomes	Interventional	Harmful
116	Male C57BL/6 mice, 1‐h tMCAO	Increased TGF‐β1 expression correlated with improved outcomes after tMCAO, at 28 DPI	Interventional	Protective
15	C57BL/6J mice (sex ND), 1‐h tMCAO	TGF‐β1 increased by CS, correlating with reduced BBB injury, increased angiogenesis, reduced mortality, and reduced infarct size	Interventional	Protective
117	Male Kunming mice, 1‐h tMCAO	Activation of TGF‐β1 by B3galt2 shown to be neuroprotective, with TGF‐β1 silencing abrogating this effect	Interventional, TGF‐β1 siRNA	Protective
118	Male SD rats, 2‐h tMCAO	Inhibition of BRD4 (modifying TGF‐β1 signaling and reducing Smad2/3 phosphorylation) reduced infarct volume and markers of fibrosis in vivo	Interventional	Harmful
119	Male C57BL/6 mice, 45‐min tMCAO	Increased correlates with improved outcomes (NSS and beam balance)	Interventional	Protective
120	C57BL/6 mice (sex ND), 90‐min tMCAO	Preventing TGF‐β1 depletion after MCAO linked with reduced BBB leakage	Interventional	Protective
121	Female Wistar rats, 2‐h tMCAO	Significantly upregulated TGF‐β1 in ischemic core and MCA smooth muscle compared with non‐MCAO controls	Observational	Protective
122	Male SD rats, 1‐h tMCAO	Significantly upregulated TGF‐β1 in brain lysate correlated with BCL‐2 upregulation	Interventional	Protective

Model, role of TGF‐β1, study type (including interventions relevant to TGF‐β1), and protective function of TGF‐β1 for all included studies modeling ischemic strokes. BBB indicates blood–brain barrier; BCL‐2, B‐cell lymphoma 2; BMSC, bone marrow stromal cell; CBF, cerebral blood flow; CSF, cerebrospinal fluid; DPI, days postinjury; HT, hemorrhagic transformation; hUS, human umbilical cord; IL‐4, interleukin 4; LRG1, leucine‐rich α‐2 glycoprotein 1; MCA, middle cerebral artery; MCAO, middle cerebral artery occlusion; MCP, monocyte chemoattractant protein; MIP, macrophage inflammatory protein; MSC, mesenchymal stem cell; NBP, n‐butylphthalide; ND, not disclosed; NSS, Neurological Severity Score; pMCAO, permanent middle cerebral artery occlusion; RMST, rhabdomyosarcoma 2–related transcript; ROS, reactive oxygen species; rt‐PA, recombinant tissue plasminogen activator; SD, Sprague–Dawley; SHR, spontaneous hypertension; siRNA, small interfering RNA; TGF‐1, transforming growth factor β1; TGF‐βRI, transforming growth factor β receptor type 1; tMCAO, transient middle cerebral artery occlusion; TSP4, thrombospondin‐4; UK, urinary kallidinogenase; and ZEB1, zinc finger E‐box binding homeobox 1.

Fourteen ischemic stroke studies were performed with no intervention, typically examining TGF‐β1 protein or RNA concentrations in brain tissue or exploring the role of components of the TGF‐β pathway in functional recovery. An increase in TGF‐β1 expression was noted specifically in the cortex,^80^, ^87^ ischemic core,^121^ cervical spinal cord,^109^ and hypometabolic zones^94^ following MCAO. An increase in TGF‐β1 levels or activation was shown to improve the number of NG2+ microglia^53^ and CD68+ microglia/macrophages^108^; increase neuroprotection,^77^ angiogenesis,^14^ and vascular remodeling^85^; and correlate with spontaneous recovery.^82^, ^97^, ^100^ In addition, suppression of the TGF‐β1/Smad3 pathway by miR‐323 was shown to increase apoptosis after stroke.^68^

Only 24 ischemic stroke studies (36%) modulated TGF‐β1 directly, either by giving exogenous TGF‐β1/overexpressing TGF‐β1 (13 studies) or inhibiting TGF‐β1/receptor (14 studies). Exogenous TGF‐β1 was commonly used as an intervention and was shown to reduce hemorrhagic transformation (HT) (2.5 μg intravenously),^81^ limit neuronal injury (50 ng intracerebroventricular),^83^ and reduce infarct volume and motor deficit (0.25 ng intracisternal).^84^ TGF‐β1 was also shown to reduce infarct volume and apoptosis, improve neurological function (1 μg intranasally),^86^ reduce infarct volume, and improve cerebral blood flow (10/50 μg, internal carotid injection)^90^ and neurological outcome (1 ng/g)^113^ poststroke. HT was reduced by exogenous TGF‐β1 in monocyte‐/macrophage‐depleted mouse brains,^72^ and by the tyrosine kinase inhibitor imatinib, which increased TGF‐β1 expression.^75^ One study demonstrated the anti‐inflammatory effect of TGF‐β1 (50/100 ng cortical injection) in suppressing nuclear factor κβ activation but did not examine any functional outcomes.^16^ No included studies demonstrated worse functional outcomes in ischemic stroke following treatment with exogenous TGF‐β1, although TGF‐β1 antagonism was linked to reduced basement membrane fibrosis in one MCAO model study.^67^

**Figure 5:**
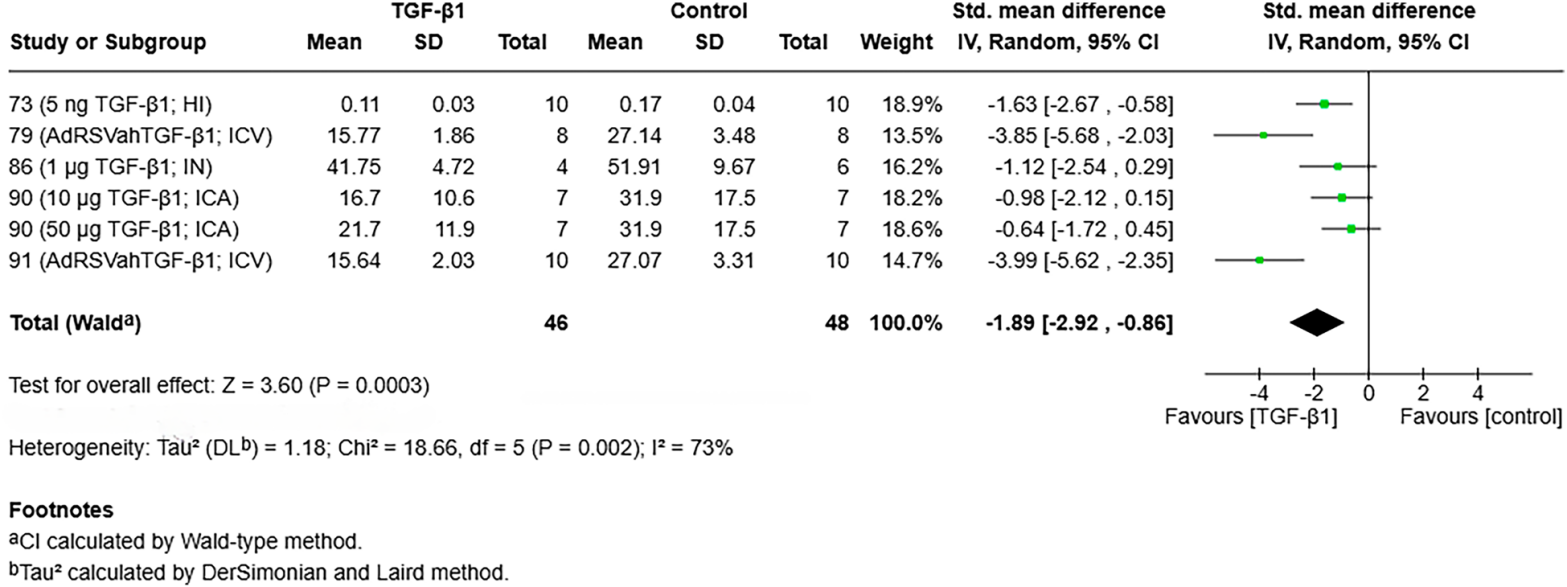
Random effects model forest plot of ischemic stroke studies measuring the effect of exogenous TGF‐β1 on infarct size. HI indicates hippocampal injection; ICA, internal carotid artery injection; ICV, intracerebroventricular; IN, intranasally; IV, inverse variance; and TGF‐β1 indicates transforming growth factor β1.

Of the 13 studies using exogenous TGF‐β1/TGF‐β1 overexpression, only 6 studies used infarct size as an end point. One of these studies did not include extractable data; therefore, a meta‐analysis was performed on 5 studies including a total of 94 animals. Analysis revealed that TGF‐β1 treatment significantly favored a reduction in infarct size in animal ischemic stroke models (random effects model: standardized mean difference, −1.62 [95% CI, −2.13 to −1.10], *I* ^2^=73%) (Figure 5; fixed effects model shown in Figure S1).

A range of anti‐inflammatory agents were employed among the cohort, with propolis,^59^ minocycline,^61^ and wogonin^62^ shown to increase TGF‐β1 levels following stroke, correlating with improved neurological outcomes or infarct volume. Intracerebroventricular injection of recombinant interleukin 4 activated TGF‐β1 signaling, reducing apoptosis and infarct volume and decreasing neurological injury.^92^ However, one embolic stroke study demonstrated that treatment with an anti‐inflammatory peptide suppressed TGF‐β1 levels, which improved neurological outcome.^101^

Genetic modification of the TGF‐β1/Smad pathway was also used as an intervention. Overexpression of microglial transcription factor zinc finger E‐box binding homeobox 1 (ZEB1) was shown to reduce infarct volume and improve blood–brain barrier (BBB) functionality in a TGF‐β1–dependent manner.^60^ Smad3 overexpression reduced apoptosis following MCAO,^63^ while TGF‐β1 overexpression reduced infarct volume^79^ and expression of the proapoptosis protein Bad.^91^ Similarly, overexpression of ALK5, a TGF‐β1 family receptor, was shown to improve neurological scoring following stroke.^112^ Transgenic inhibition of astrocyte TGF‐β1 expression generated increased cortical inflammation and infarct size, in addition to worsened neurological severity.^96^ However, modification of the TGF‐β signaling pathway did show a correlation between increased TGF‐β1 expression and worsened outcomes in some studies. Overexpression of leucine‐rich α‐2 glycoprotein 1 (LRG1) was noted to upregulate ALK1, a TGF‐β family receptor, which correlated with worsened infarct size and neurological deficits in a 1‐hour transient MCAO model.^107^ Similarly, knockdown of long noncoding RNAs MEG3^78^ and rhabdomyosarcoma 2–related transcript^64^ was shown to reduce the expression of TGF‐β1 and key proinflammatory cytokines, correlating with improved outcomes. Adenoviral knockdown of BRD4 modified TGF‐β1 signaling, reducing phosphorylation of Smad2/3 and resulting in improved infarct size and reduced fibrosis.^118^

Antagonistic inhibition of TGF‐β1 receptors was used in several studies, revealing TGF‐β1–dependent reductions in infarct volume following ischemic stroke.^73^, ^74^, ^88^ Inhibiting TGF‐β receptor type 2 was shown to result in increased HT in a neonatal MCAO model.^103^ However, one study showed a link between ALK5 inhibition and reduced reactive oxygen species damage, correlating with improved outcomes after MCAO. In addition, one study showed a correlation between TGF‐β1 expression and increased fibrosis and reduced perivascular CSF flow in the subacute stage of the injury (7–14 days postinjury).^83^

Postinjury conditioning was investigated with a range of stimuli. Isoflurane postconditioning (for 60 minutes immediately after MCAO) was shown to reduce Neurological Severity Score (NSS), infarct volume, and neuronal survival or density in a TGF‐β1–dependent manner.^66^, ^69^, ^105^ Remote ischemic postconditioning generated an increase in TGF‐β1 expression and phosphorylation of Smad2/3, which correlated with reduced infarct volume and an improved NSS, confirmed by TGF‐β1 knockdown.^76^ Hypoxic postconditioning, in which animals were contained in a 5% O_2_ atmosphere for 45 minutes, was shown to increase ALK5 expression, correlating with improvement in the NSS.^119^

Cell therapies were also investigated. Mesenchymal stem cell transplantation was shown to reduce infarct volume^98^ and improve NSS^106^ in a TGF‐β–dependent manner, and to increase serum TGF‐β1 levels^99^ when administered intravenously. Dental pulp stem cell injection also upregulated the TGF‐β1 pathway, which correlated with reduced infarct size and an improved NSS.^102^ Inversely, one study showed a downregulation of TGF‐β1 following mesenchymal stem cell injection (intravenously), although the effect of this on functional outcomes was not shown.^89^

A wide range of other interventions were studied, with a majority showing a link between increased TGF‐β1 expression and positive outcomes. Extracellular vesicles derived from oxygen‐ and glucose‐deprived cells upregulated the TGF‐β1 pathway, improving NSS.^93^ Cerebral laser therapy (660 nm, 2.64 J/cm^2^) was shown to upregulate TGF‐β1 in the serum, which correlated with reduced nitric oxide synthase expression and was suggested to reduce the generation of cytotoxic nitric oxide.^95^ TGF‐β1 and the Smad signaling pathway were upregulated by urinary kallidinogenase,^65^ the neuroprotective agent N‐butylphtalide,^70^ bovine myelin basic protein,^71^ tamoxifen,^110^ vitamin D3,^114^ R,S‐ketamine,^116^ coicis semen,^15^ nicotine^120^ and β‐1, 3‐galactosyltransferase 2,^117^ all improving outcomes including infarct size and neurological function. Electroacupuncture was shown to increase TGF‐β1 expression following transient MCAO, correlated with increased expression of the apoptosis suppressor BCL2.^122^ However, the opposite was shown in 3 studies. A reduction in TGF‐β1 expression or signaling—achieved by pretreatment with efonidipine,^115^ 2 Chinese herbal medicines,^111^ or ALK5 inhibitors^104^—was correlated with improved outcome scores.

#### TGF‐β1 in Preclinical Hemorrhagic Stroke Studies

Table 2, ^54^, ^55^, ^56^, ^57^, ^58^, ^123^, ^124^, ^125^, ^126^, ^127^, ^128^, ^129^, ^130^, ^131^, ^132^, ^133^, ^134^, ^135^, ^136^, ^137^, ^138^ details the model, role of TGF‐β1, study type, and outcome for each included hemorrhagic stroke study.

**Table 2:** Hemorrhagic Stroke Study Characteristics

Reference #	Model	Role of TGF‐β1	Study type	TGF‐β1 role
123	Male SD rats, EP of ICA	TGF‐β1 promotes brain injury postcerebral hemorrhage	Interventional	Harmful
124	Wistar rat pups, intracerebroventricular injection of 80 μL, high hematocrit adult rat blood	No effect on TGF‐β1 seen	Interventional, including TGF‐β1 antagonist decorin	Unclear
125	C57BL/6 mouse pups, intrathecal injection of 30 μL serum, plasma or TGF‐β1 with leakage into SAS	Induced hydrocephalus, both as injected hrTGF‐β1 and as TGF‐β1 in serum	Interventional, anti‐TGF‐β1 antibody	Harmful
126	Male SD rats, EP of MCA	TGF‐β1 significantly increased with CB2R agonist treatment	Interventional	Protective
127	Male C57BL/6 or db/db mice, collagenase injection (0.075 U) into basal ganglia 1 μL of 10 ng/μL TGF‐β1 injected intrastriatally 10 min before ICH induction	TGF‐β1 administration abolished the protective effects of adiponectin	Interventional, including exogenous TGF‐β1	Harmful
54	Male SD rats, autologous blood intracisternal injection	Decorin reduced TGF‐β1/pSmad2/3 and CTGF, improving neurological outcomes and preventing ventricular enlargement from SAH	Interventional, including TGF‐β1 antagonist decorin	Harmful
128	Male SD rats, EP of ICA	Protective against SAH	Interventional	Protective
55	Male SD rats, autologous blood intracisternal injection	Reduction in TGF‐β1 expression in tissue and CSF correlates with reduced hydrocephalus and reduced CTGF expression	Interventional	Harmful
129	Male mice, transgenic B6.SJL‐Ptprca Pep3b/BoyJ (CD45.1), C57/BL6J (WT), and B6.129P‐Cx3cr1tm1Litt/J (Cx3cr1GFP/GFP and Cx3cr1+/GFP), injected with whole blood or collagenase (0.05 U)	Suggested to reduce proinflammatory cytokine production	Interventional, exogenous TGF‐β1	Protective
56	Male SD rats, intracisternal injection of autologous blood (0.5 mL)	Reduction in TGF‐β1 correlated with improved outcomes and reduced hydrocephalus following SAH	Interventional	Harmful
130	Male C57BL/6J mice, EP of circle of Willis	Increased TGF‐β1 post‐SAH	Observational	Harmful
131	Wistar rat pups, intracerebroventricular injection of rat blood or artificial CSF	TGF‐β1 expression seems to correlate with development of posthemorrhagic hydrocephalus	Observational	Harmful
132	Male C57BL/6 mice, collagenase (0.3 U) into basal ganglia	TGF‐β1 improves survival, reduces BBB damage, and exerts anti‐inflammatory effects	Interventional, exogenous TGF‐β1	Protective
58	New Zealand white rabbit pups, glycerol intracerebroventricular injection	TGF‐β1 reduced following IVH and rescued with USSCs	Interventional	Protective
133	Male C57BL/6 mice, injection of 10 μL autologous blood (2.5 mm deep to surface)	TGF‐β1 does not contribute to mouse ICH severity	Observational	Unclear
134	Wistar rat pups, intracerebroventricular injection of 80 μL high hematocrit blood	Oral anti–TGF‐β drugs had no effect on IVH	Interventional, oral anti–TGF‐β1 drugs	Unclear
135	SD rat pups, collagenase (0.3 U) intracerebroventricular injection	Inhibiting TGF‐βRI reduced pSMAD2/3, development delay, NSS, brain atrophy, and ventricular dilation when used at 60 mg/kg compared with control	Interventional, including TGF‐βRI inhibitor	Harmful
57	Male SD rats, intracerebroventricular injection of 200 μL autologous blood	Reduced TGF‐β1 expression at 14 DPI correlated with reduced SAS/ventricular wall fibrosis and improved neurological outcomes	Interventional	Harmful
136	Male piglets, right frontal injection of autologous blood (1 mL followed by 1.5 mL 5 min later)	Minocycline may exert a protective effect by upregulating TGF‐β1	Interventional	Protective
137	Male CD‐1 mice, intracerebral injection of collagenase (0.075 U)	TGF‐β1 pathways activation by AHR signaling may worsen outcome post‐ICH	Interventional	Harmful
138	Male CD‐1 mice, intrastriatal injection of autologous blood (30 μL)	Increased TGF‐β1 expression and pSmad2/3 increased neurological outcomes post‐ICH, abrogated by administering TGF‐β1 antagonist	Interventional, including TGF‐β1 inhibitor	Protective

Model, role of TGF‐β1, study type (including interventions relevant to TGF‐β1), and protective function of TGF‐β1 for all included studies modeling hemorrhagic strokes. AHR indicates aryl hydrocarbon receptor; BBB, blood–brain barrier; CBR2, cannabinoid receptor 2; CSF, cerebrospinal fluid; CTGF, connective tissue growth factor; DPI, days postinjury; EP, endovascular perforation; hrTGF‐β1 human recombinant transforming growth factor; ICA, internal carotid artery; ICH, intracerebral hemorrhage; MCA, middle cerebral artery; NSS, Neurological Severity Score; SAH, subarachnoid hemorrhage; SAS, subarachnoid space; SD, Sprague–Dawley; TGF‐β1, transforming growth factor β1; TGF‐βRI, transforming growth factor β receptor type 1; USSC, unrestricted somatic stem cell; and WT, wild‐type.

**Figure 6:**
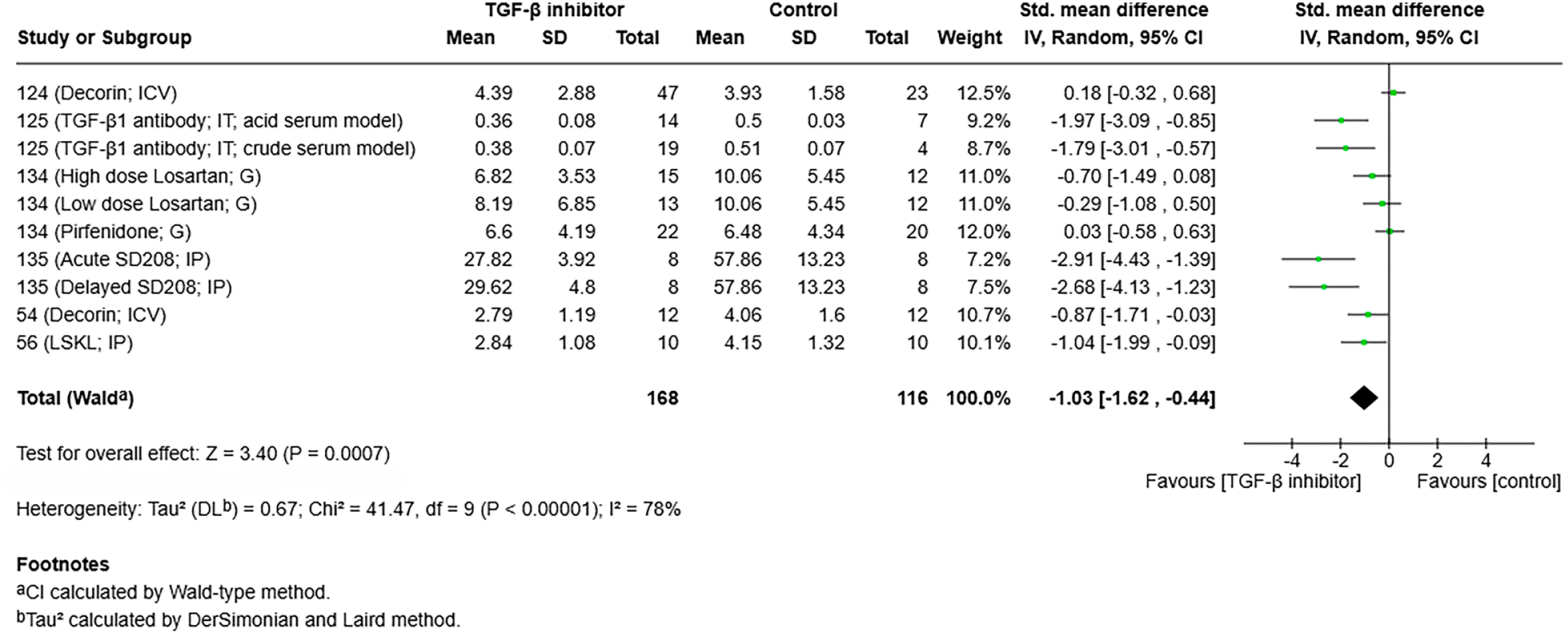
Random effects model forest plot of hemorrhagic stroke studies measuring the effect of TGF‐β1 inhibitors or antagonists on ventricular size. G indicates oral gavage; ICV, intracerebroventricular; IP, intraperitoneal; IT, intrathecal; IV, inverse variance; and TGF‐β1, transforming growth factor β1.

Three hemorrhagic stroke studies were performed without intervention. Of these, 2 suggested involvement of TGF‐β1 in hemorrhagic stroke; showing that TGF‐β1 was increased in a model of SAH^130^ and that TGF‐β1 expression correlated with the development of posthemorrhagic hydrocephalus following IVH.^131^ Furthermore, one study reported the induction of hydrocephalus following intrathecal injection of 6 ng TGF‐β1, or TGF‐β1–containing serum.^125^ Ten hemorrhagic stroke studies (48%) modulated TGF‐β1 directly with 3 studies delivering exogenous TGF‐β1 (3 ICH studies) and 7 studies directly applying TGF‐β1 antagonists or inhibitors (6 studies in SAH/GMH using anti–TGF‐β1 antibodies, decorin, LSKL, SD208, pirfenidone, and losartan; 1 study in ICH using SB431542). Most studies involving TGF‐β1 inhibition/antagonism reported significantly improved NSS and reduced ventricular enlargement.^54^, ^56^, ^124^, ^125^, ^135^ However, the role of TGF‐β1 antagonism with the oral anti–TGF‐β1 drugs pirfenidone and losartan was unclear, with no effect noted in an IVH model.^134^ A meta‐analysis was performed on the 6 SAH/GMH studies that used ventricular size as an end point, which included a total of 284 animals. Analysis revealed that TGF‐β1 inhibition/antagonism significantly favored a reduction in ventricular size in animal hemorrhagic stroke models (random effect model: standardized mean difference, −1.03 [95% CI, −1.62 to –0.44]; *I* ^2^=78%) (Figure 6; fixed‐effect model shown in Figure S2). These studies suggested a link between inhibiting TGF‐β1 and improved outcome following hemorrhagic stroke, typically by preventing fibrosis and hydrocephalus development, especially in SAH models.

Exogenous TGF‐β1 (3 studies) was given only as an intervention in ICH models and showed some protective effects in 2 studies, with reduced NSS and BBB leakage reported following injection of TGF‐β1 to the striatum^129^ and peritoneum.^132^

The cannabinoid receptor 2 (CBR2) agonist JWH‐133 generated increased TGF‐β1 expression and reduced NSS and brain water content in an SAH model.^126^ Interestingly, the opposite effect was noted in an IVH model, with JWH‐133 being shown to downregulate TGF‐β1 and improve functional outcomes while reducing subarachnoid fibrosis and lateral ventricle size.^57^

Simvastatin, a 3‐hydroxy‐3‐methylglutaryl‐coenzyme A reductase inhibitor, also exerted a protective effect, with a reduced NSS and higher expression of TGF‐β1 in the brainstem.^128^ Minocycline was also shown to exert a protective effect in ICH models, reducing cerebral edema and neurological deficit and correlating with increased TGF‐β1 at the transcript level.^136^ Unrestricted somatic stem cell intracerebroventricular infusion was also investigated. It was found to exert a protective effect by increasing TGF‐β1 protein in the CSF and RNA in the brain, which was associated with increased cell proliferation.^58^ TGF‐β1 was, however, suggested to not contribute to severity of ICH in one mouse model.^133^

Inversely, TGF‐β1 expression correlated with negative outcomes in studies of the antioxidant resveratrol,^123^ the insulin sensitizer adiponectin,^127^ and an aryl hydrocarbon receptor agonist.^137^ Colchicine^124^ generated no significant effect on TGF‐β1 expression or relevant outcomes.

### RoB in Preclinical Stroke Studies

**Figure 7:**
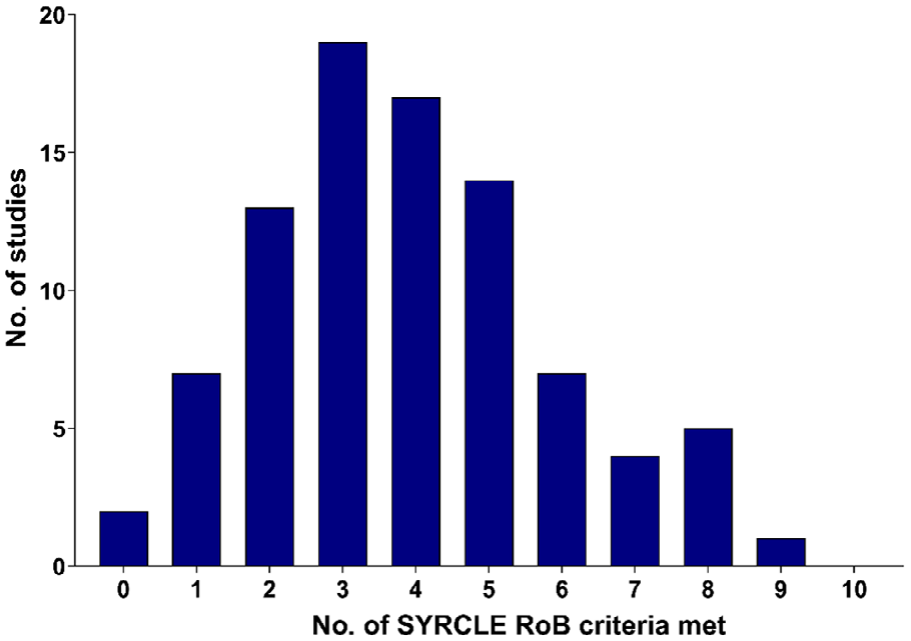
Histogram of the number of SYRCLE RoB criteria met by included studies. RoB indicates risk of bias; and SYRCLE, Systematic Review Centre for Laboratory Animal Experimentation.

The studies included in the present review typically showed low compliance with the SYRCLE RoB tool for preclinical animal studies, with a low score indicating a high RoB. The mean number of SYRCLE RoB criteria met was 3.90 among 89 studies, with 2 studies agreeing with zero of the criteria, and no studies complying with all 10 criteria (Figure 7). Complete data showing the total RoB score for each study included in the present work is shown in Table S5.

## DISCUSSION

This review reveals the wide variety of models and methodological approaches used among preclinical studies of stroke. A majority of ischemic stroke studies used the Longa MCAO model (or modifications thereof), as this enables craniectomy‐free, reversible induction of cerebral ischemia.^139^ However, the ischemia time was highly variable, as shown in Figure 2, although this did not appear to correlate with a change in the role of TGF‐β1 in these models. Previous works have highlighted a difference in inflammatory responses between the Longa MCAO method and an alternative, the Koizumi method,^140^ suggesting that methodology plays a large role in the outcome of the study. The variety of methods was even wider in hemorrhagic strokes, with studies modeling ICH, IVH, SAH, and GMH in different ways. Standardization of the methodological approach may be necessary to ensure that results obtained are both true effects and comparable between studies.

Furthermore, nearly all studies in the present review used male animals or mixed unsexed pups. A total of 5 studies of the included 89 used female animals, with one controlling for the estrus cycle.^121^ It has previously been demonstrated that estrogen levels affect the sensitivity of female rats to ischemic stroke,^141^ with estrogen suggested to be neuroprotective even in male rats.^142^ Indeed, human studies have shown a markedly greater risk of stroke after menopause when estrogen levels decline.^143^ A failure of preclinical studies to control for this important variable may limit the utility of such studies, drawing conclusions that may only apply to the male sex. RoB was generally high within the included studies, as shown in Figure 5, with notably low compliance with randomization and investigator blinding. However, the SYRCLE RoB tool used to assess these studies has only been available in the past 10 years,^22^ with many studies in this review conducted before this period. Further universal adoption of a preclinical study bias tool may be highly beneficial, improving confidence that the results are bias‐free.

This review demonstrates that TGF‐β1 likely exerts a protective, beneficial effect in ischemic stroke and a more detrimental effect in hemorrhagic stroke. In ischemic stroke, the main beneficial effects of TGF‐β1 were on oxidative stress/inflammation, blood vessels/BBB, and apoptosis/neuroprotection. Studies that demonstrated TGF‐β1 had detrimental effects in ischemic stroke were focused on fibrosis and inflammation. Similar negative effects of TGF‐β1 in IVH and SAH models were observed, mainly associated with fibrosis and the development of hydrocephalus. ICH studies were mixed, with TGF‐β1 reducing inflammation but increasing apoptosis and affecting the BBB. However, it should be noted that only 33 of the 89 studies directly modulated TGF‐β1 (exogenous or inhibiters) and therefore the majority of studies only demonstrated associations or correlations relating to TGF‐β1.

Increased activation of the TGF‐β1 pathway was noted in all ischemic stroke studies performed without intervention, correlated with improvement in a range of therapeutic outcome measures. TGF‐β1 was shown to be involved in the production of new blood vessels^14^ and vascular remodeling,^85^ with previous work identifying the role of TGF‐β1 signaling in enhancing neovessel formation.^144^ This angiogenic function of TGF‐β1 may aid in improving blood flow to ischemic tissues and enabling recovery of function in the subacute phase of the injury, as was noted in multiple studies. TGF‐β1 was also shown to limit HT.^72^, ^75^, ^81^ HT is a common, severe complication of ischemic stroke, wherein the BBB is sufficiently disrupted by poststroke inflammation as to allow the extravasation of blood from the circulation into the brain tissues.^145^ Therefore TGF‐β1 has utility in reducing both the primary effect of the ischemic stroke (tissue ischemia and reduction in cerebral blood flow) and possible sequalae, including HT.

Many studies of ischemic stroke administered exogenous TGF‐β1 to examine this effect further. Remarkably, all included studies employing exogenous TGF‐β1 showed improved outcome measures compared with control groups, regardless of dosage or route. A meta‐analysis (Figure 5) further supported this finding, with the pooled effect measure of studies employing exogenous TGF‐β1 or TGF‐β1 overexpression showing a significant reduction in infarct size in treated animals. Heterogeneity was found to be significant between these studies, suggesting substantial variability between the included studies, although this is an expected finding due to the included studies employing a range of doses or administration routes for TGF‐β1 or overexpression vectors.

Intranasal administration and injection into the cisterna magna, ventricles, carotid artery, and cortex all yielded improvements in key functional outcome measures, including NSS and infarct volume. Proinflammatory cytokines and oxidative stress markers were frequently and significantly reduced following stroke by a range of anti‐inflammatory agents, which correlated with increased TGF‐β1 levels. Treatment of inflammation has previously been suggested as a therapeutic target following ischemic stroke, with the aim of limiting neuronal damage and improving neurological recovery after stroke.^146^ Apoptosis was also limited by TGF‐β1 administration or upregulation, with reduced apoptotic markers and infarct areas noted in several studies.

Similar results were reported in studies examining the effect of exogenous cytokines and in some studies employing genetic modification to overexpress TGF‐β1 or members of the TGF‐β1 signaling pathway. In addition, 3 studies found that inhibition of TGF‐β1 signaling correlated with worsened outcomes.^73^, ^74^, ^88^

However, it should be noted that some studies, particularly those examining TGF‐β1/Smad overexpression by genetic modification, showed a correlation to worsened outcome following stroke.^64^, ^78^, ^107^, ^118^ Astrocytic TGF‐β1 overexpression has previously been linked to glial activation, hydrocephalus, and amyloid deposition.^147^ It is suggested that overexpression approaches may generate sustained, abnormally high TGF‐β1 levels or activation, which persist far beyond that found in a normal inflammatory response. One study identified that raised TGF‐β1 levels in the subacute phase of ischemic stroke model led to cortical fibrosis and reduced glymphatic CSF outflow.^67^ TGF‐β1 is widely implicated in fibrosis, with high levels triggering the deposition of excessive, disordered extracellular matrix in wound healing models.^148^, ^149^, ^150^ Timing may indeed be crucial when considering the use of TGF‐β1 as a therapeutic in ischemic stroke, as delivery too late after the injury may fail to limit primary effects (infarct size, apoptosis, HT) while increasing the risk of fibrosis and worsening the outcome. Further work may aid in identifying the optimal timepoint for administering exogenous TGF‐β1 or TGF‐β1–activating therapies in ischemic stroke.

While the present review suggests that TGF‐β1 may be beneficial in ischemic stroke, preclinical hemorrhagic stroke studies suggest that TGF‐β1 may worsen the outcome, although this picture is more nuanced than in ischemic stroke. Antagonism or inhibition of TGF‐β1 signaling was generally shown to correlate with an improvement in outcome,^54^, ^55^, ^56^, ^135^, ^138^ typically assessed by NSS and ventricular enlargement, a marker of hydrocephalus. Hydrocephalus has previously been generated and modeled by overexpression of TGF‐β1, both in stroke studies^131^ and congenital hydrocephalus research^151^ and by intrathecal injection of exogenous TGF‐β1.^125^ It is hypothesized that, in hemorrhagic stroke, TGF‐β1 may play a predominantly profibrotic role, compared with the therapeutic role seen in ischemic stroke. One study within the present review examined the role of CBR2 in regulating TGF‐β1 levels after IVH, finding that downregulation of TGF‐β1 led to reduced fibrosis of the subarachnoid space.^57^ Subarachnoid fibrosis has previously been implicated in the generation of posthemorrhagic hydrocephalus, reducing CSF outflow via the arachnoid granulations and glymphatics in a TGF‐β1–dependent manner.^152^ Reducing TGF‐β1 levels following hemorrhagic stroke may reduce morbidity caused by hydrocephalus, ultimately increasing quality of life.

As previously discussed, all ischemic stroke studies that administered exogenous TGF‐β1 showed an improvement in outcome, and the majority of studies investigating TGF‐β1 antagonism or signaling inhibition demonstrated worsened outcomes if TGF‐β1 function was interrupted. Inversely, most hemorrhagic stroke studies that directly applied TGF‐β1 antagonists or inhibitors reported improvements in outcome in models of SAH, ICH, and GMH. A meta‐analysis (Figure 6) also supported the utility of TGF‐β1 inhibitors or antagonists in animal models of hemorrhagic stroke, with a pooled effect measure indicating significantly reduced ventricular size in treated animals. Heterogeneity was found to be high and significant, although this is again an expected finding due to the diverse range of models, animal species, treatments, and routes used.

A limitation of the present study is that all assessed hemorrhagic stroke studies were grouped together, while the pathophysiology of ICH, IVH/GMH, and SAH were markedly different. This may explain the nuanced role of TGF‐β1 in hemorrhagic stroke in the current review, although the number of included studies does not provide enough evidence to perform further analysis based on subtype of hemorrhagic stroke. Further work may explore the role of TGF‐β1 within different subtypes of hemorrhagic stroke to elucidate its function. However, this does not limit the utility of comparisons between hemorrhagic and ischemic stroke, marked by the blockage of blood flow and by the presence of blood in the tissue or CSF‐filled spaces, respectively.

While it is apparent that the role of TGF‐β1 differs between ischemic and hemorrhagic strokes, the mechanism underlying this difference has not been directly investigated. It is hypothesized that the nature of the inflammatory stimulus is likely the determining factor in the role of TGF‐β1 in stroke. Blood and its degradation products are widely recognized as cytotoxic to brain tissue,^10^ exacerbating the effects of stroke beyond the physical disruption caused by the blood volume.^153^ For this reason, surgical and pharmacological hematoma clearance have been investigated as treatment following hemorrhagic stroke to reduce the incidence of morbidity caused by secondary brain injury.^154^, ^155^, ^156^ In the present review, observational preclinical ischemic stroke studies typically demonstrated an early peak in TGF‐β1 expression, often at 3^14^ or 7^16^, ^77^, ^85^ days postinjury, followed by a slightly raised expression into the subacute phase of the injury.^82^, ^109^ Studies typically indicated a 2‐fold^157^ to 3‐fold^67^, ^85^ increase in TGF‐β1 expression or signaling between sham and MCAO‐injured animals.

In hemorrhagic stroke studies, however, elevated TGF‐β1 levels were recorded in the CSF at 14^131^ to 21^54^ days postinjury, with one study recording TGF‐β1 levels 10‐fold greater than control at 14 days postinjury.^158^ It is hypothesized that the timing, location, and magnitude of TGF‐β1 expression and signaling may determine the role of the molecule. The more sustained cytotoxic insult caused by blood products following hemorrhagic stroke may cause a more continuous release of TGF‐β1, at a much higher level than that seen following ischemic stroke. This may result in a shift away from the possible healing properties of TGF‐β1 seen in ischemic stroke, towards a profibrotic role commonly seen in hemorrhagic strokes. This hypothesis is supported by evidence showing that persistent or repeated TGF‐β1 expression and activation correlated with fibrosis severity in liver disease^159^ and pancreatitis.^160^ This effect may also explain the observed negative effects of TGF‐β1 overexpression in ischemic studies,^64^, ^78^, ^107^, ^118^ with this prolonged upregulation surpassing that typically seen following ischemic stroke.

**Figure 8:**
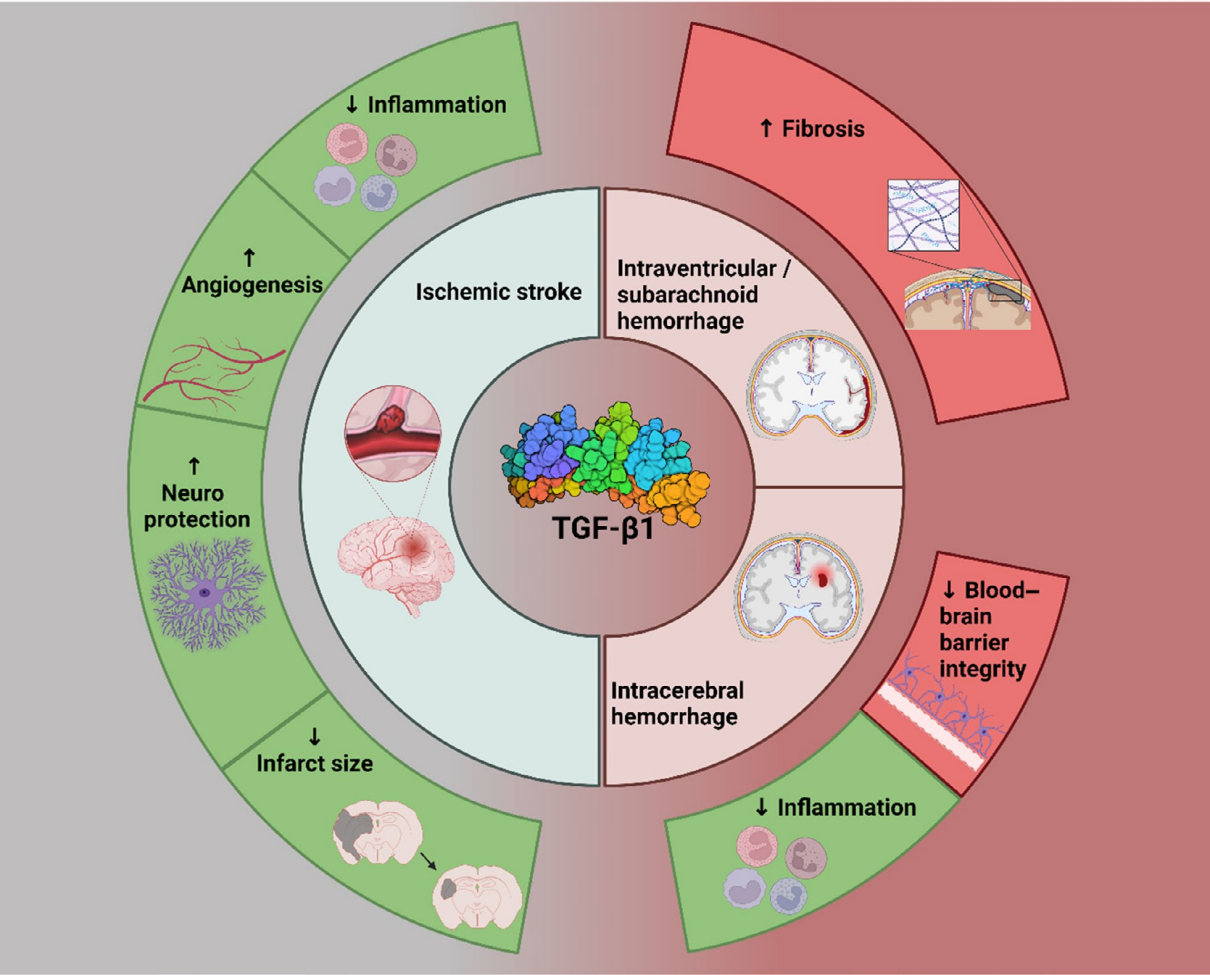
Outline of proposed differential TGF‐β1 function in hemorrhagic stroke and ischemic stroke. TGF‐β1 indicates transforming growth factor β1. Created with BioRender.com.

As summarized in Figure 8, the present systematic review demonstrates that TGF‐β1 appears to play a largely neuroprotective, anti‐inflammatory role in ischemic stroke. Notably, exogenous TGF‐β1 was shown to improve measured outcomes in all studies utilizing this approach. However, TGF‐β1 has been shown to potentially exacerbate fibrosis and hydrocephalus in hemorrhagic stroke. It is hypothesized that the release of blood into the subarachnoid space, ventricles, and brain tissues by hemorrhagic strokes may generate a more sustained and higher level of TGF‐β1 expression than seen in ischemic strokes. This continuously high level of TGF‐β1 may shift the role of the molecule from healing towards a profibrotic role, increasing the incidence of secondary brain injury and additional morbidity. Further studies to directly compare both the magnitude and time course of TGF‐β1 expression or signaling in different types of stroke may aid in confirming or rejecting this hypothesis. This review also demonstrates the clinical importance of TGF‐β1 in stroke, showing that locally applied TGF‐β1 inhibitors may improve outcomes in hemorrhagic stroke, although this effect was less clear than the marked utility of exogenous TGF‐β1 treatment following ischemic stroke. Suitable delivery and timing of TGF‐β1–based therapies may aid in reducing both primary and secondary brain injury, reducing poststroke morbidity.

## Sources of Funding

This research was primarily funded by Wellcome (ISSF Continuity Award) and the Medical Research Foundation (MRF‐076‐0002‐RG‐BOTF‐C0754).

## Disclosures

None.

## Supporting information

Tables S1–S5
Figures S1–S2
