# Supplementary material for: Systematic Review of the Differential Effects of TGF‐β1 in Ischemic and Hemorrhagic Preclinical Stroke Models
Source: J Am Heart Assoc. 2025 Jul 1;14(14):e037890. doi: 10.1161/JAHA.124.037890 (PMC12533622; doi:10.1161/JAHA.124.037890)

SUPPLEMENTAL MATERIAL

Table S1. PRISMA declarations.

Section and Topic	Item #	Checklist item	Location where item is reported
**TITLE**			Page
Title	1	Identify the report as a systematic review.	1
**ABSTRACT**			
Abstract	2	See the PRISMA 2020 for Abstracts checklist.	2
**INTRODUCTION**			
Rationale	3	Describe the rationale for the review in the context of existing knowledge.	6/7
Objectives	4	Provide an explicit statement of the objective(s) or question(s) the review addresses.	7
**METHODS**			
Eligibility criteria	5	Specify the inclusion and exclusion criteria for the review and how studies were grouped for the syntheses.	9/10
Information sources	6	Specify all databases, registers, websites, organisations, reference lists and other sources searched or consulted to identify studies. Specify the date when each source was last searched or consulted.	8
Search strategy	7	Present the full search strategies for all databases, registers and websites, including any filters and limits used.	8
Selection process	8	Specify the methods used to decide whether a study met the inclusion criteria of the review, including how many reviewers screened each record and each report retrieved, whether they worked independently, and if applicable, details of automation tools used in the process.	9-11
Data collection process	9	Specify the methods used to collect data from reports, including how many reviewers collected data from each report, whether they worked independently, any processes for obtaining or confirming data from study investigators, and if applicable, details of automation tools used in the process.	9-11
Data items	10a	List and define all outcomes for which data were sought. Specify whether all results that were compatible with each outcome domain in each study were sought (e.g. for all measures, time points, analyses), and if not, the methods used to decide which results to collect.	9-11
	10b	List and define all other variables for which data were sought (e.g. participant and intervention characteristics, funding sources). Describe any assumptions made about any missing or unclear information.	9-11
Study risk of bias assessment	11	Specify the methods used to assess risk of bias in the included studies, including details of the tool(s) used, how many reviewers assessed each study and whether they worked independently, and if applicable, details of automation tools used in the process.	9
Effect measures	12	Specify for each outcome the effect measure(s) (e.g. risk ratio, mean difference) used in the synthesis or presentation of results.	11
Synthesis methods	13a	Describe the processes used to decide which studies were eligible for each synthesis (e.g. tabulating the study intervention characteristics and comparing against the planned groups for each synthesis (item #5)).	10/11
	13b	Describe any methods required to prepare the data for presentation or synthesis, such as handling of missing summary statistics, or data	10/11

Section and Topic	Item #	Checklist item	Location where item is reported
		conversions.	
	13c	Describe any methods used to tabulate or visually display results of individual studies and syntheses.	10/11
	13d	Describe any methods used to synthesize results and provide a rationale for the choice(s). If meta-analysis was performed, describe the model(s), method(s) to identify the presence and extent of statistical heterogeneity, and software package(s) used.	10/11
	13e	Describe any methods used to explore possible causes of heterogeneity among study results (e.g. subgroup analysis, meta-regression).	10/11
	13f	Describe any sensitivity analyses conducted to assess robustness of the synthesized results.	10/11
Reporting bias assessment	14	Describe any methods used to assess risk of bias due to missing results in a synthesis (arising from reporting biases).	9
Certainty assessment	15	Describe any methods used to assess certainty (or confidence) in the body of evidence for an outcome.	N/A
**RESULTS**			
Study selection	16a	Describe the results of the search and selection process, from the number of records identified in the search to the number of studies included in the review, ideally using a flow diagram.	12
	16b	Cite studies that might appear to meet the inclusion criteria, but which were excluded, and explain why they were excluded.	12
Study characteristics	17	Cite each included study and present its characteristics.	Table 1 and 2
Risk of bias in studies	18	Present assessments of risk of bias for each included study.	Table S4
Results of individual studies	19	For all outcomes, present, for each study: (a) summary statistics for each group (where appropriate) and (b) an effect estimate and its precision (e.g. confidence/credible interval), ideally using structured tables or plots.	Figure 5 and 6
Results of syntheses	20a	For each synthesis, briefly summarise the characteristics and risk of bias among contributing studies.	Table 1 and 2, Table S2, S3, S4
	20b	Present results of all statistical syntheses conducted. If meta-analysis was done, present for each the summary estimate and its precision (e.g. confidence/credible interval) and measures of statistical heterogeneity. If comparing groups, describe the direction of the effect.	Figure 5 and 6
	20c	Present results of all investigations of possible causes of heterogeneity among study results.	Ischemic – Treatment, route and species shown in Figure 2 and 3, discussed on page 34.

Section and Topic	Item #	Checklist item	Location where item is reported
			Hemorrhagic – treatment and route discussed on page 26
	20d	Present results of all sensitivity analyses conducted to assess the robustness of the synthesized results.	Figure S1 and S2
Reporting biases	21	Present assessments of risk of bias due to missing results (arising from reporting biases) for each synthesis assessed.	Table S4
Certainty of evidence	22	Present assessments of certainty (or confidence) in the body of evidence for each outcome assessed.	N/A
**DISCUSSION**			
Discussion	23a	Provide a general interpretation of the results in the context of other evidence.	22-28
	23b	Discuss any limitations of the evidence included in the review.	26
	23c	Discuss any limitations of the review processes used.	26
	23d	Discuss implications of the results for practice, policy, and future research.	27-28
**OTHER INFORMATION**			
Registration and protocol	24a	Provide registration information for the review, including register name and registration number, or state that the review was not registered.	8
	24b	Indicate where the review protocol can be accessed, or state that a protocol was not prepared.	8
	24c	Describe and explain any amendments to information provided at registration or in the protocol.	N/A
Support	25	Describe sources of financial or non-financial support for the review, and the role of the funders or sponsors in the review.	29
Competing interests	26	Declare any competing interests of review authors.	29
Availability of data, code and other materials	27	Report which of the following are publicly available and where they can be found: template data collection forms; data extracted from included studies; data used for all analyses; analytic code; any other materials used in the review.	Available in supplemental material

Table S2. Ischemic stroke study characteristics. Animal species, model, experimental groups, intervention and reported outcomes for all included studies modelling ischemic strokes.

tMCAO = transient middle cerebral artery occlusion (ischemia / reperfusion), SD = Sprague-Dawley, SHR = spontaneous hypertension, ICV = intracerebroventricular, IV = intravenous, DMSO = dimethyl sulfoxide, PO = per os, IP = intraperitoneal, DPI = days post-injury, CBF = cerebral blood flow, VEGF = vascular endothelial growth factor, αSMA = alpha smooth muscle actin, NSS = neurological severity score, BBB = blood brain barrier, BWC = brain water content, ISH = in-situ hybridization, IHC = immunohistochemistry.

**Study**	**Model**	**Experimental groups**	**Intervention**	**Outcome summary**	**What/how measured**	**Summary of role of TGF-β1**	**TGF-β1 role**
(Abdel-Rahman *et al.*, 2020) [59]	Male Wistar rats, two-hour tMCAO .	12 animals per group; sham, tMCAO control, tMCAO + 50 mg/kg propolis, tMCAO + 100 mg/kg propolis.	Propolis delivered by gavage at 50 and 100 mg/kg for 10 days, started 7 days prior to tMCAO.	Propolis reduced oxidative stress, reduced neurological deficits and improved behavioral assessments. Propolis increased gene expression of TGF-β1 and reduced MMP-9 gene expression.	TGF-β1 mRNA by qPCR in the brain.	TGF-β1 was increased following ischemic stroke. Propolis increased TGF-β1 levels further, suggesting protective role.	Protective.
(Li *et al.*, 2018) [60]	Male C57BL/6 mice, tMCAO (time not disclosed).	6 animals per group; wild type, ZEB1 overexpression and ZEB1 knockdown.	Overexpression or knockdown of ZEB1. Intracerebral injection of AAV-shTGFBRII ($6x10^9$ IU), three weeks prior to tMCAO.	tMCAO significantly increased ZEB1 expression in ischemic hemisphere. ZEB1 overexpression reduced infarct volume and Evans blue extravasation. Knockdown with AAV-shTGFBRII removed this protective effect.	TGF-β1 protein by IHC in penumbra.	Activation of TGF-β1 signaling pathway by ZEB1 reduces infarct volume and maintains BBB.	Protective.
(Yang *et al.*, 2015) [61]	Male SHR rats, 90-minute tMCAO.	8 animals per group; vehicle control and minocycline treated.	Minocycline, 3 mg/kg IV, single dose immediately after MCAO. Vehicle 10% DMSO in solutrol.	Minocycline reduced infarct size and prevented tissue loss at 2-4 weeks post injury, correlated with decreased MMP2 and 3 and improved BBB. Minocycline decreased TNFα and IL1β, while increasing TGF-β1.	TGFβ protein by IHC and WB in brain.	TGF-β1 increased by minocycline treatment, correlating with improved outcomes.	Protective.
(Kong *et al.*, 2019) [62]	Male SD rats, pMCAO by embolus injection.	20 animals per group; sham, tMCAO control, tMCAO + wogonin, substitutes.	Wogonin, dissolved at 20 mg/100ml in 0.9%NaCl. Animals given 10 ml/kg/day, controls	Significant reduction in neurological deficits between ischemic and wogonin-treated animals at 14 DPI. Significant increase in vascular density in peri-infarct region with	TGF-β1 protein by WB in ischemic brain.	TGF-β1 increase correlated with reduction in NSS and increase in vascular density.	Protective.

			given same volume normal saline. Both given orally once a day for 14 consecutive days.	wogonin compared to sham and ischemic group. All correlated with significantly increased in TGF-β1 protein levels in ischemic tissue following wogonin treatment.			
(Liu *et al.*, 2015) [63]	Male SD rats, two-hour tMCAO.	10 animals per group; sham and control. Five animals per group; control, vector and SMAD3 over expression – repeated across four timepoints.	SMAD3 overexpression by AAV9-SMAD3 ($3x10^{13}$ vg/ml/kg), three days prior to tMCAO.	pSMAD2 and pSMAD significantly increased at 6, 24, 72 hours and 7 days post-MCAO compared to sham<br><br>Upregulation of SMAD3 resulted in reduced cleaved caspase 3 expression, indicating reduced apoptosis.	Smad2/3 mRNA by pPCR in penumbra. P-Smad2/3 and Smad2/3 protein by WB in penumbra.	Upregulation of TGF-β1 pathway leads to reduced apoptosis markers after MCAO.	Protective.
(J. Li *et al.*, 2022) [64]	Male BALB/c mice, 20-minute tMCAO.	18 animals per group; sham, tMCAO, siRNA-RMST, siRNA-RMST negative control, miR-221-3p, miR negative control, RMST overexpression (OE) + miR-221-3p, RMST OE + miR negative control, RMST OE + interference of PIK3R1, RMST OE + interference negative control.	Nucleotide sequences injected by ICV to lateral ventricle in artificial CSF. Sequences at 100 µMol/L, 1.5 µl, injected 24 hours prior to MCAO.	Inhibiting RMST attenuates brain injury post-MCAO (BWC) and reduces inflammation and ox stress (TNFα, IL6, IL1b). siRMST reduces TGF-β1 protein levels, correlated with improved outcomes, via miR-221-3P. Functional outcomes (Neurological score, rotarod, forelimb grip) all improved with injection of miR-221-3p mimic.	TGF-β1 protein by WB in brain.	Reduced TGF-β1 activation by suppressing RMST with miR-221-3p resulted in better outcomes.	Harmful
(Dong *et al.*, 2016) [65]	Male SD rats, two-hour tMCAO.	36 animals per group; blank control, treatment and Gegen control. Further divided across five timepoints; 12 animals at 72 hours post injury, six animals at	Treatment group and Gegen control groups injected IV with UK ($8.75x10^{-3}$ PNAU/kg) and pueratin (100 mg/kg) 30 mins post-MCAO	Treatment with UK resulted in reduction in infarct volume compared to control. All groups saw increases in TGF-β1 positive cells over time post-MCAO. Significant increase in TGF-β1 positive cells in animals	TGF-β1 protein by IHC in cortex.	Increased in expression by MCAO, but also significantly increased by treatment with UK, correlating with reduced infarct volume.	Protective.

		each successive timepoint.	induction. Control injected with same volume of saline.	treated with UK compared to control.			
(G. X. Zhang *et al.*, 2019) [66]	Male SD rats, 90-minute tMCAO.	12 animals per group; sham, tMCAO, tMCAO + isoflurane (ISO), tMCAO + ISO + Dkk-1, tMCAO + Dkk-1, tMCAO + ISO + LY2157299, tMCAO + ISO + SIS3, tMCAO + ISO + LY2157299 + SIS3.	Isoflurane post-conditioning (1.5% for 60 minutes following reperfusion), Dkk-1 (Wnt inhibitor) 5 µg/kg ICV 30 minutes prior to tMCAO, LY2157299 (TGFβ inhibitor), SIS3 (Smad3 inhibitor).	Isoflurane postconditioning reduced infarct volume, NSS and neuron apoptosis, while also increasing VEGF, Wnt3a, bCatenin and reducing Caspase 3.<br>Inhibiting TGF-β1 / Smad3 with LY2157299 decreased bCatenin in nucleus and cytoplasm, suggesting involvement of TGF-β1 in this pathway.	TGF-β1, Smad3, p-Smad3 protein by WB in hippocampus and cortex.	Inhibiting TGF-β1 reduced markers of isoflurane post-conditioning, which had been shown to reduce NSS, infarct volume and neuronal apoptosis.	Protective.
(Howe *et al.*, 2019) [67]	Male C57BL/6 mice, permanent MCAO via cauterisation. Young (3 month old) or aged (20 month old).	Animal numbers given as ranges, reuse between experiments unclear, but between 70 to 125 young and aged animals used.<br>5-8 animals per group, 4 groups; young (sham and MCAO), aged (sham and MCAO).<br>4-5 per group, 3 groups, for fibrosis studies (naïve, vehicle and TGFb1 inhibitor)<br>4-5 per group for vehicle and TGF-β1 injection. 5-8 per group, 6 groups) for OGD vs NO studies (split into vehicle, TGF-β1 and amyloid-β.	TGFβ receptor antagonist (GW788388) 10mg/kg/day via osmotic pump for 7 days from 7 DPI, motor function assessed at 14 DPI.<br>rTGF-β1 injected intracisternally (3 ng/ml).	MCAO increased perivascular GFAP and lectin in aged rats. Also increased cortical GFAP, vimentin and TGF-β1 in same conditions for both young and aged rats.<br>Infusion of TGF-β1 receptor antagonist resulted in reduced perivascular and cortical GFAP and fibronectin expression compared to vehicle control.	TGF-β1 protein by ELISA in cortex.	TGF-β1 worsens fibrosis and neurological recovery and modifies perivascular CSF flow in aged mice MCAO model.	Harmful.

(Che *et al.*, 2019) [68]	Male SD rats, two-hour tMCAO.	6 animals per group; control and tMCAO.	No intervention.	MicroRNA-323 expression upregulated in infarct group vs control, suggested to correlate with nerve cell apoptosis.<br>In vitro studies show that SMAD3 exp. was reduced with miR-323 overexpression	Smad3 mRNA by gene chip array.	Suppression of TGF-β1/SMAD3 by miR-323 increased neurotoxicity in cerebral infarct model.	Protective.
(Yin *et al.*, 2020) [69]	Male SD rats. 90-minute tMCAO.	8 animals per group; sham, tMCAO, tMCAO + ISO, tMCAO + LY2157299, tMCAO + LY2157299 + ISO, tMCAO + Ro318220, tMCAO + Ro318220 = ISO, tMCAO + 18β-GA, tMCAO + 18β-GA+1.5% ISO, tMCAO + ISO + LY2157299 + Ro318220, DMSO, and ISO+DMSO.	Isoflurane post-conditioning (1.5% for 60 minutes following reperfusion),<br>All drugs injected ICV (lateral ventricle), 30 minutes prior to tMCAO at 5 µg/kg (20 µl), in 0.5% DMSO;<br>TGF-β1 inhibitor LY2157299,<br>p-Cx43 inhibitor,<br>p-Cx43 activator 18β-GA.	Isoflurane postconditioning increased viable neuron density and reduced infarct volume and NSS post-MCAO, correlated with increased TGF-β1 (mRNA and protein) and ratio of pSmad2/3:Smad2/3 with ISO+MCAO vs MCAO control.<br>TGF-β1 protein expression and viable neuron density reduced in ISO+MCAO+TGF-β1 inhibitor groups vs ISO+MCAO alone.	TGF-β1 mRNA by pPCR in hippocampus.<br>TGF-β1, smad2/3, p-Smad2/3 protein by WB in hippocampus.	Increased TGF-β1 expression following isoflurane post-conditioning correlates with reduced NSS, reduced infarct volume and increased viable neuron density.	Protective.
(Jiang *et al.*, 2016) [70]	Male SD rats, tMCAO for 6 hours, 1 day and two days.	10 animals sham operated, 30 animals each for NBP treatment and vehicle control.	25 mg/kg N-Butylphthalide (NBP) PO twice a day.	VEGF and TGF-β1 upregulated compared to sham. Slightly greater TGF-β1 protein levels 1 day post-MCAO in NBP treated group than MCAO control, correlated with improved neuro score and decreased infarct size on DPI 1 and 2.	TGF-β1 protein by IHC and mRNA by RT-PCR in the brain.	TGF-β1 slightly upregulated by NBP, correlated with improved NSS and infarct size.	Protective.
(Becker *et al.*, 2003) [71]	Male Lewis rats, three-hour tMCAO.	tMCAO group split into 12 receiving myelin basic protein (MBP) treatment and 14 ovalbumin control.	Tolerized to bovine MBP or OVA (control) by 5 nasal administrations over 2 weeks. 100	Significant increase in TGF-β1 secreting cells in MCAO brains in recipients of MBP-tolerized cells.<br>MBP-tolerized animals secreted more TGF-β1 in response to MBP	TGF-β1 protein by ELISPOT in mononuclear cells from the	Increased secretion of TGF-β1 correlates with reduced infarct size.	Protective.

			µg antigen in 40 µl PBS	than control animals, combined with significant reduction in infarct volume in MBP-tolerized animals.	brain in each group.		
(Sugimoto *et al.*, 2014) [53]	Male Wistar rats, 90-minute tMCAO.	Total N not given	No intervention	Expression of NG2+ microglia in demarcation region, possible as a result of ischemia. NG2 expression previously shown to be induced by TGF-β1<br>Marked increase in TGF-β1 and TGFBRI/II mRNA in ischemic core vs contralateral side, with smaller increase in peri-ischemic tissue	TGF-β1, 2 and 3 and TGFβR1 and R2 mRNA by qPCR in contralateral, peri-infarct and core.	Increase in TGF-β1 post stroke may increase number of NG2+ microglia, which may improve outcome	Protective.
(Gliem *et al.*, 2012) [72]	Male C57BL/6 mice, both wild type and transgenic – (CD45.1)(B6.SJL-PtprcaPepcb/BoyJ), (CCR2-/-)(B6.129S4-Ccr2tm1Ifc/J) and (CX3CR1-/-(B6.129P-Cx3cr1tm1Litt/J)). 40-minute tMCAO or cortical photothrombosis.	9 animals per group; MO/MP depletion.<br>4 animals per group for gene expression studies post-photothrombosis, 6 animals per group for TGF-β1 injection studies.	MO/MP depletion performed with clodronate (7 mg/ml of liposomes).<br>TGF-β1 (25 ng in 2 µl PBS) injected 2mm lateral, caudal, medial and rostral to coordinates of photothrombosis on days 0, 1 and 2 post-injury	MO/MP depleted mice showed hemorrhagic infarct transformation at sig. greater rate than PBS control. Photothrombosis in WT mice caused marked increase in TGF-β1 expression compared to control at DPI4. Reduction in TGF-β1 expression from WT to MO/MP depleted mice, both at gene and protein level.<br>TGF-β1 injection into MO/MP depleted brains after photothrombosis but prior to hemorrhagic transformation lead to reduction in bleeding, partially corrected neovessel morphology and increase in pSmad2 in infarct border	TGF-β1 and thrombospondin-1 mRNA by qPCR in brain.<br>TGF-β1 and pSmad2 protein by ICH in the brain.	TGF-β1 reduced hemorrhagic transformation in MO/MP depleted brains and corrected neovessel morphology	Protective.
(Zhu *et al.*, 2017) [73]	Male SD rats, 90-minute tMCAO	10 animals per group; healthy, sham, tMCAO, tMCAO + TGF-β1, tMCAO + TGF-β1 inhibitor	TGF-β1 or TGF-β1 inhibitor (0.1 µg/ml, 50µl) injected into hippocampus after MCAO	TGF-β1 treatment reduced infarct size compared to the MCAO control, and group injected with TGF-β1 inhibitor.<br>Immunohistochemistry and western blotting revealed increase in TGF-β1/Smad3 pathway	TGF-β1 and Smad3 protein by IHC and WB, and mRNA by RT-PCR in brain.	Increased TGF-β1 expression and Smad3 activation correlated with reduced infarct size	Protective.

(Yu *et al.*, 2018) [74]	Male albino Wistar rats, 20-minute tMCAO	6 animals per group; control, placebo, tMCAO, tMCAO + TGF-β1, tMCAO + TGF-β1 inhibitor	Un-named TGF-β1 inhibitor injected IP (100 µl) after tMCAO. No information on dosage and timing.	TGF-β1 supplementation decreased infarct size, whilst addition of TGF-β1 inhibitor abrogated this effect, producing a greater mean infarct size than the MCAO only group	TGF-β1 and Smad3 protein by IHC in brain.	TGF-β1 supplementation reduced infarct size post-MCAO.	Protective.
(Nguyen *et al.*, 2021) [75]	Male mice (species not disclosed), 45-minute tMCAO or combined cortical photothrombosis and proximal MCA ligation	Total N not given.<br>N=4-7 per group for Fig 1 (PT MCAO)<br>N=6-8 per group for Fig 2<br>N=3 for 2 groups for Fig 3	Imatinib dissolved in DMSO at 100 µg/ml, 200 mg/kg/mouse, PO. Acute phase group - Imatinib at 1 and 8 hours post-MCAO up to 3 days post-injury, 3-5 day post injury for sub-acute group.	Decreased TGF-β1 expression correlated with increased hemorrhagic transformation	TGF-β1 protein by IHC in lesion.	Increased TGF-β1 levels reduced hemorrhagic transformation in ischemic stroke models	Protective.
(Ma *et al.*, 2024) [76]	Male SD rats, two-hour tMCAO	12 animals per group; sham, tMCAO, remote ischemic postconditioning (RIP), in-situ ischemic postconditioning, TGF-β1 knockdown	In-situ ischemic postconditioning - clamping bilateral carotid for 10 seconds, release for 30 seconds x3 cycles.<br>remote ischemic postconditioning - clamping femoral arteries for 10 min, release 10 mins x3 cycles.<br>TGF-β1 siRNA knockdown.	TGF-β1 RNA upregulated post-MCAO compared with sham. RIP resulted in downregulated TGF-β1 RNA, but upregulated protein.<br>RIP significantly reduced infarct volume and apoptosis in penumbra compared to MCAO control, in addition to improved neurological scores<br>RIP significantly reduced Smad3 expression compared to MCAO control	TGF-β1 and Smad2/3 protein by IHC and WB. p-Smad2/3 and Smad4 protein by WB. TGF-β1 Smad2/3 and Smad4 mRNA by qPCR. All in penumbra. TGF-β1 and Smad3 protein by ELISA in serum.	Increase in TGF-β1 protein and p-Smad2/3 correlated with improved outcomes	Protective.
(Lehrmann *et al.*, 1998) [77]	Male SHR rats, one-hour tMCAO	2 animals per tMCAO group; 6 hours, 1, 3, 7, 21 days or 3 months). 2 animals sham.	No intervention	TGF-β1 expression increased at timepoints up to 7DPI. TGF-β1 mRNA mainly expressed by microglia and macrophages	TGF-β1 mRNA by ISH in brain.	Suggested neuroprotective effect when taking primary data	Protective.

						with other literature in discussion	
(Long *et al.*, 2023) [78]	Male SD rats, permanent MCAO.	12 animals sham, 10 MCAO, 10 low expression MCAO.	AAV knockdown of MEG3.	Knockdown of MEG3 resulted in reduced infarct volume, reduced neuro deficits, reduced IL-1β, TNFα and increased IL-10 24 hours after MCAO.<br>Reduced TUNEL positive cells and Caspase 3 expression following MEG3 knockdown, in addition to reduced TGF-β1 protein expression and Smad2/3 ost-MCAO	TGF-β1, Smad2/3 protein by WB in cerebral tissue.	Decreased TGF-β1 and Smad2/3 expression reduced pro-inflammatory signaling and improved outcomes.	Harmful.
(Pang *et al.*, 2001) [79]	Male CD-1 mice, 30-minute tMCAO.	5-7 animals per group; control, 0, 1, 3 and 7 DPI.	Overexpression of TGF-β1 five days prior to MCAO by ICV injection of AAV-TGF-β1, 1 µl into lateral ventricle at 0.2ul/min. Saline control.	TGF-β1 overexpression led to reduced infarct area DPI 1, 3 and 7. Significant increase in monocyte chemoattractant protein-1 (MCP) and macrophage inflammatory protein-1α (MIP) in infarct hemisphere at DPI 1 and 3 in TGF-β1 overexpression vs control.	TGF-β1 protein by ELISA in brain.	TGF-β1 overexpression reduced infarct volume and increased MIP and MCP expression.	Protective.
(Meng *et al.*, 2016) [14]	Male SD rats, permanent MCAO.	6 animals per group; control, 2, 6, 12 hours post injury, 1, 3, 7, 14 DPI.	No intervention	LRG1 and TGF-β1 RNA and protein expression significantly increased at 12 h post-MCAO, peaking at 3 DPI. Correlated with neovascularisation in ischemic rat brain.	TGF-β1 protein by IHC and WB and mRNA by qPCR in penumbra.	TGF-β1 protein increased post-MCAO, correlated with increased neovascularisation	Protective.
(Vincze *et al.*, 2010) [80]	Male Wistar rats, one-hour tMCAO	14 animals total.	No intervention.	FISH used to show high TGF-β1 expression in cortex post-MCAO.	TGF-β1, 2 and 3 mRNA by ISH in brain.	TGF-β1 increased post-MCAO, especially in cortex.	Unclear.
(Cai *et al.*, 2015) [81]	Male SD rats, autologous clot injection into internal carotid artery.	13 animals sham, 23 control, 21 for both MCAO + rt-PA and MCAO +rt-PA + TGF-β1.	rt-PA at 10mg/kg, 10% bolus, 90% infusion over 30 mins, IV. 3h post-MCAO.<br>TGF-β1 (2.5 µg), IV, with rt-PA.	Significant increase in hemorrhage and Evans blue extravasation (marker of BBB failure) with rt-PA treatment compared to control, which was reduced to near control level with rt-PA+TGF-β1. rt-PA significantly reduced expression of	TGF-β1 not measured.	TGF-β1 protects against hemorrhagic transformation induced by rt-PA treatment post-thromboembolic MCAO.	Protective.

				key BBB basement membrane + tight junction proteins (IHC), which was rescued in rt-PA + TGF-β1 group. PAI1 expression sig. reduced by rt-PA, rescued in rt-PA + TGF-β1 group.<br>MMP-2+9 activity increased in rt-PA group vs control, and sig. decreased in rt-PA+TGF-β1 group.			
(Wang *et al.*, 1995) [82]	Male SHR rats, permanent MCAO.	4 animals per group; sham, 1, 3, 6 and 12 hours post injury, 1, 2, 5, 10 and 15 DPI.	No intervention.	Increase in TGF-β1 mRNA expression over time post-MCAO, significant at 2, 5, 10 and 15 days.	TGF-β1 mRNA by RT-PCR in contralateral and ipsilateral.	TGF-β1 increased in sub-acute stage of stroke, suggesting link to healing processes.	Protective.
(Henrich-Noack, Prehn and Krieglstein, 1996) [83]	Male Wistar rats, systemic hypotension combined with bilateral carotid artery occlusion, 10-minute ischemic time.	ICV TGF-β1 experiments; 9 animals TGF-β1 group, 11 vehicle control, 12 animals 50 ng TGF-β1 group (repeated in further 9 animals). Hippocampal TGF-β1 injection experiments; 6 animals vehicle control, 8 animals 4 ng TGF-β1.	TGF-β1 in 2 µl of vehicle (saline +0.1%BSA) given either intraventricularly or into hippocampus tissue 1 hr prior to ischemia.	TGF-β1 has the capacity to reduce injury to CA1 hippocampal neurons caused by transient global ischemia in rats. Possible stabilization of neuronal Ca^{2+} homeostasis.	TGF-β1 not measured.	Reduces injury to hippocampal neurons and stabilizes neuron Ca^{2+} homeostasis.	Protective.
(Lin *et al.*, 2016) [84]	Male SD rats, 90-minute tMCAO.	Total N not given, number varies throughout figures.	Intracisternal injection of 25µl neuron conditioned culture medium (NCM) or TGF-β1/GDNF/NT-3/ ERK agonist (10 ng/ml). Interventions given at end of tMCAO, animals culled 24 h later.	Neuron conditioned medium (NCM) following glucose, oxygen and serum starvation (mimicking ischemia) contained more TGF-β1 than normal control media.<br>Injection of TGF-β1 caused significant reduction in infarct volume and motor deficit compared to MCAO control. Same effect noted with injection of NCM, which contained elevated TGF-β1.	TGF-β1 not measured.	TGF-β1 injection reduced infarct volume and motor deficit following MCAO.	Protective.

(Buscemi *et al.*, 2019) [85]	Male C57BL/6J mice, 30-minute tMCAO.	3 animals per group (3 and 12 hours post injury), 6 animals per group (control, 24 hours, 48 hours and 1 week post injury).	No intervention	TGF-β1 increases over time post-MCAO, as does astrocyte reactivity (GFAP). Both correlate with vascular remodeling and stromal reaction post-MCAO.	TGF-β1 protein by IHC in the lesion area over timepoints.	TGF-β1 increased vascular remodeling and tissue repair.	Protective.
(Islam *et al.*, 2018) [16]	Male Wistar rats, 90-minute tMCAO.	4 animals per group for TGF-β1 injection experiments.	Injection of TGF-β1 (50 or 100 ng) into ischemic cortex at lesion site, 48 hours post-injury in 1.25 and 2.5 µl saline.	Increase in RNA expression of TGF-β1in ischemic core compared to control at 7 DPI, correlating with increased TGF-β1 protein expression at same timepoint.	TGF-β1 mRNA by qPCR and TGF-β1 protein by WB in the contra, peri, core.	Increased expression in ischemic core at 7 DPI by tMCAO.	Unclear.
(Ma *et al.*, 2008) [86]	Male C57BL/6 mice, 90-minute tMCAO.	30 animals control, 20 TGF-β1 group.	TGF-β1 given intranasally (20 µl, 1 µg TGF-β1), two hours post-MCAO. Second series 24 hours post-MCAO.	Neurological function was greater post-MCAO in TGF-β1-treated group than control.<br>Infarct volume lower after MCAO in TGF-β1 treated group than control (DPI 7, 14, 21 and 28)<br>TGF-β1 treatment reduced apoptosis 7 days post-MCAO compared with control.<br>BrdU-incorporation and NeuN expression increased in TGF-β1 group at DPI28, indicating neurogenesis.	TGF-β1 not measured.	Intranasal TGF-β1 reduces infarct volume, improves neuro function and reduced apoptosis.	Protective.
(Pál *et al.*, 2012) [87]	Male Wistar rats. Either one-hour tMCAO or permanent MCAO.	5 animals each for analysis at 3, 24 and 72 hours, and 1 month post tMCAO<br>5 animals for analysis at 24 hours post permanent MCAO<br>plus 11 sham operated rats (2 per timepoint and 3 additional for perfusion at 24 and 72	No intervention.	Significant increase in TGF-β1 protein level in lesion-side cortical layers ii-v, and in caudate putamen.<br>Increase in TGF-β1 in area around lesion 3 h post-tMCAO.<br>More marked increase in TGF-β1 following permanent MCAO than with any tMCAO timepoint. TGF-β1 induced in glial cells but not neurons at 72 h post-tMCAO,	TGF-β1, 2 and 3 mRNA by ISH around lesion area.	Induced rapidly post-tMCAO, even in regions which don't express TGF-β1 when un-injured.	Unclear.

		hours and 1 month post tMCAO..					
(Ruocco *et al.*, 1999) [88]	Male SD rats, 30-minute tMCAO.	4 animals vehicle, 6 NMDA, 6 NMDA + TGF-β1, 6 NMDA + TBRIIs-Fc, 3 TBRIIs-Fc only, 6 MCAO + saline, 6 MCAO + TBRIIs-Fc, 3 sham + TBRIIs-Fc.	Intracortical injection of TBRIIs-Fc (1.5 µg) 30 mins prior to MCAO.<br>NMDA (75 nmol) combined with 3 ng TGF-β1 or 1.5 µg TBRIIs-Fc, or TBRIIs-Fc alone.	Antagonising TGF-β1 significantly increased lesion size in NMDA-induced injury, whilst injection of TGF-β1 significantly reduced it.<br>In MCAO-induced injury, antagonism of TGF-β1 resulted in an increase in lesion size.	TGF-β1 mRNA by RT-PCR in striatum and cortex ipsilateral and contralateral.	Antagonism of TGF-β1 increases size of excitotoxicity- and MCAO-induced lesions.	Protective.
(Xin *et al.*, 2013) [89]	Male C57BL/6J mice, permanent MCAO.	9 animals per group; control or MSC injection.	IV Injection of mouse-derived MSCs 24 hours post-MCAO.	MSC treatment downregulated TGF-β1 expression and pSmad2/3 in the ischemic boundary zone 14 days post-MCAO. Double staining showed that this downregulation occured in both astrocytes and microglia / macrophages.	TGF-β1 and p-Smad2/3 protein by IHC in lesion.	TGF-β1 expression downregulated post-MCAO by MSC treatment.	Unclear.
(Gross *et al.*, 1993) [90]	Male and female New Zealand white rabbits, autologous clot delivery to internal carotid artery.	7 animals per group; control, 10 µg TGF-β1, 50 µg TGF-β1.	TGF-β1 (10 or 50 µg in 50 µl of 4mM HCl, delivered to internal carotid immediately before ischemia induction.	10 µg TGF-β1 increased CBF vs control at 10, 60 and 120 minutes post-stroke. 10 µg TGF-β1 significantly reduced infarct size compared to control. 50 µg TGF-β1 produced no effect and appeared to reduce CBF.	TGF-β1 not measured.	Reduced infarct size after 4 hours of ischemia, and increased CBF after 10, 60 and 120 minutes.	Protective.
(Zhu *et al.*, 2002) [91]	Male CD-1 mice, 30-minute tMCAO.	3 animals for sham and tMCAO control. 13-20 animals for AdRSVahTGF-β1, AdRSVlacZ and saline control groups.	$1x10^{12}$ particles of AdRSVahTGF-β1(induces overexpression of TGF-β1), or same dose of AdRSVlacZ (Control adenovirus).	TGF-β1 activates MAPK pathway, causing phosphorylation of Bad, preventing apoptosis. TGF-β1 overexpression reduces Bad levels post-MCAO, corresponding with reduced ischemic volume, improved neurological scoring and reduced DNA damage.	Induced TGF-β1 protein by ELISA and IHC in brain. P-ERK1/2 protein by IHC in brain.	TGF-β1 may suppress pro-apoptotic protein Bad, correlated with improved outcomes .	Protective.

(Huang *et al.*, 2023) [92]	Male SD rats, two-hour tMCAO.	5 groups; normal (only used for western blot studies), sham, tMCAO at 3, 7, 14 and 21 DPI. 3 animals per group for western blotting, 6 for IOD measurements, 6 for macrophage infiltration studies, 3 for scar formation.	ICV injection of PBS, DMSO (10 µl), rrIL4 (800ng/d in 10 µl PBS) or cyclopamine (20 µM). All at 0.5ul/h for 7 or 14 days, starting 2 h post-MCAO.	Marked fibrosis in ischemic core post-MCAO, reduced by macrophage depletion with Cyclopamine. Treatment with IL4 post-MCAO upregulates TGF-β1 expression in tissue at DPI 3 and 7, correlated with reduced infarct volume, apoptosis and neurological score vs MCAO control at DPI7.	TGF-β1 protein by WB in ischemic core.	TGF-β1 activation by IL4 reduces infarct volume, apoptosis and NSS.	Protective.
(L. Zhang *et al.*, 2021) [93]	Male C57BL/6 mice, one-hour tMCAO.	N = 75 total animals	IV injection of extracellular vesicles from oxygen-glucose deprivation preconditioned primary microglia – 10 µg in 200 µl PBS) at the onset of reperfusion and 6 hours post-MCAO.	EV treatment upregulated TGF-β1/Smad2/3 pathway at protein level, correlated with improved functional recovery and neurological scoring. Knockdown of TGF-β1 with siRNA reversed this effect.	TGF-β1, pSmad2/3 and Smad2.3 protein by WB in ischemic hemisphere.	TGF-β1 / Smad2/3 pathway upregulation reduced neurological deficits post-MCAO.	Protective.
(Ali *et al.*, 2001) [94]	Male baboons, permanent MCAO.	3 animals per group; 1 and 7 days MCAO.	No intervention.	TGF-β1 expression is increased in baboon brains post-MCAO, primarily localized to zones of hypometabolism.	TGF-β1 mRNA by RT-PCR and protein by WB in healthy and lesioned tissue.	TGF-β1 expression is increased following ischemic stroke.	Unclear.
(Leung *et al.*, 2002) [95]	Male SD rats, one-hour tMCAO.	Total of 94 animals used, no breakdown of number per experimental group provided.	Laser (660 nm, 2.64 J/cm^2, 10 kHz) applied to cerebrum for 1, 5 or 10 minutes immediately post-MCAO.	NOS levels increased at DPI 4, 5 and 6 after tMCAO without treatment. 1-, 5- and 10-minute laser exposure reduced NOS levels post-MCAO, corresponding with increased serum TGF-β1 protein expression.	TGF-β1 protein by WB in brain.	TGF-β1 upregulated by laser treatment, correlating with reduced NOS expression.	Protective.
(Cekanaviciute *et al.*, 2014) [96]	Female transgenic (Ast-Tbr2DN)	Transgenic mouse studies; 4 animals for	Mice modified with Ast-Tbr2DN	TGF-β1 specifically inhibited in astrocytes of chosen transgenic	pSmad2 and Akt protein by IHC	Reduces inflammation in peri-infarct cortex,	Protective.

	mice, cortical photothrombosis.	wild type and 4Ast-Tbr2DN.<br>6-12 animals per group for inflammation studies following injury, 5-6 animals for cluster analysis after injury.	transgene to reduce TGF-β1 signaling.	mice. Inhibiting astrocyte TGF-β1 expression resulted in increased inflammation in peri-infarct cortex post-MCAO, in addition to larger infarct area and worsened motor function.	and WB in peri-infarct cortex TGF-β1 and thrombospondin-1 protein by Luminex in cortex.	reduces infarct size and improves motor outcomes post stroke.	
(Liu *et al.*, 2013) [97]	Male Wistar rats, permanent MCAO by autologous clot delivery to MCA.	6 animals per group, 9 groups; 0, 3, 6, 12, 24, 48, 72, 120, 168 hours post tMCAO.	No intervention.	Infarct volume was seen to spontaneously recover after 24 hours. TGF-β1 pathway was upregulated in later phase, correlated with recovery.	TGF-β1 and Smad4 mRNA by PCR	TGF-β1 pathway upregulation was correlated with spontaneous recovery from MCAO.	Protective.
(Yoo *et al.*, 2013) [98]	Male SD rats, two-hour tMCAO.	11 animals sham. 11 tMCAO + vehicle, 18 tMCAO + MSCs, 15 tMCAO + MSCs + shTGF-β1.	MSC or MSC/shTGF-β1 transplantation ($5x10^5$ cells) injected into penumbra ipsilateral to injury at DPI3.	MSC transplantation reduced infarct volume, neurological scoring and immune cell infiltration relative to MCAO control.<br>MSC-shTGF-β1 transplantation removed this effect, with immune cell migration noted but no improvement to infarct volume.	TGF-β1 protein by IHC in brain.	TGF-β1 knockdown correlated with worsened infarct volume and NSS / functional recovery.	Protective.
(Cheng *et al.*, 2015) [99]	Male mice (species not disclosed), 90-minute tMCAO	Unclear overall numbers. N=8 / group for cytokine studies, inc. TGF-β1.	IV injection of human umbilical cord MSCs	hUS-MSCs shown to significantly increase serum TGF-β1 levels post-MCAO.	TGFβ protein by ELISA in blood serum.	Increased serum TGF-β1 levels by hUS-MSCs suggested to be neuroprotective.	Protective.
(Pál, Lovas and Dobolyi, 2014) [100]	Male Wistar rats, either one-hour tMCAO or permanent MCAO.	6 animals per group, studied at: 24 hours post tMCAO or post permanent MCAO, and studies at 72 hours or 1 month post tMCAO.<br>6 sham operated rats, 2 per timepoint.<br>Additional 4 animals perfusion fixed for immunohistochemistry at each timepoint.	No intervention.	Increase in expression of TGFBRI and RII within penumbra, from very low expression at 24 hours post injury to high expression at 72 h. Infarct volume decreased over time. TGFBRI remained highly expressed up to 1 month post tMCAO, suggesting contribution to recovery.	TGFβR1, R2, R3 and ALK1 mRNA by ISH around lesion site.	TGF-β1 receptor expression is induced after tMCAO and may contribute to recovery.	Protective.

(Zhang *et al.*, 2014) [101]	Male Wistar rats, permanent MCAO.	12 animals per group; control, AcSDKP, or saline + tPA. 13 animals AcSDKP + tPA. 6 animals per group also given mAb-Ac or control antibody	Intra-arterial AcSDKP, 0.8mg/kg/day, for 3 days, starting at 1 or 4h post-MCAO, followed by subcutaneous infusion with osmotic pump for 3 days.<br>tPA 10mg/kg, IV, starting 4 h post-MCAO.<br>mAb-Ac (neutralising monoclonal antibody), 0.4 mg/kg	AcSDKP treatment post stroke had neuroprotective effects post-MCAO, decreased the levels of TGF-β1 and pSmad2/3 compared to controls.	TGF-β1 protein by IHC and WB in brain.	Suppression of TGF-β1 may improve neurological outcome in embolic stroke model	Harmful.
(Song *et al.*, 2017) [102]	Male SD rats, two hour-tMCAO.	9 animals per group; control, hDPSCs or hBM-MSCs.	Human dental pulp stem cells (hDPSCs) or human bone marrow-derived mesenchymal stem cells (hBM-MSC) IV 24 hours post-MCAO.	hDPSC injection reduced infarct volume and improved neurological deficits 28 days post-MCAO. Similar but smaller trend following hBM-MSCs.<br>TGF-β1 signaling pathway was upregulated at the gene level in both groups compared to MCAO control.	TGF-β1 not measured.	TGF-β1 pathway upregulation correlated with reduced infarct volume and improved NSS 28 days post-MCAO.	Protective.
(Fernández-López *et al.*, 2016) [103]	Unsexed rat pups and transgenic (Cx3cr1(GFP+) /Ccr2(RFP+)) mice.	Number of animals not disclosed.	Depletion of microglia by intracerebral injection of liposomes containing clodronate at p5.<br>Recombinant human TGF-β1 administered at 6	Microglial depletion results in reduced BBB and modified TGF-β1/ALK5 signaling function post-tMCAO. Adding exogenous circulating TGF-β1 does not offset this effect, so activation of microglia important.<br>Inhibition of TGFBRII results in activated microglial cells results in	TGFβ protein by IHC in the brain. P-Smad2/3 and Smad2/3 protein by WB and IHC in the brain.	Prevents hemorrhagic transformation in neonatal MCAO model.	Protective.

			hours and 90 min prior to euthanasia, also at 24 h post reperfusion. 0.5mg/kg/injection, IV.	hemorrhagic transformation. No difference in injury volume.			
(Lou *et al.*, 2018) [104]	Male SD rats, two-hour tMCAO	8 animals per group; sham, tMCAO, Sb505124 and vehicle control.	Treatment with ALK5 inhibitor Sb505124 (15 mg/kg).	Inhibiting ALK5 resulted in decreased infarct volume, decreased apoptosis and inhibited morphology modification post-MCAO compared to ischemic control. Treatment with ALK5 inhibitor resulted in reduced ALK5 and TGF-β1 levels and reduction in oxidative stress markers NOX2/4.	TGF-β1 protein by ELISA in brain tissue. ALK5 protein by WB and mRNA by qPCR. Smad2/3 protein by western blot in the brain.	Reduction in TGF-β1 levels (and ALK5 levels) led to reduction in ROS damage and thus reduced effects of tMCAO.	Harmful.
(Wang *et al.*, 2016) [105]	Male SD rats, 90-minute tMCAO.	12 animals per group; sham, tMCAO, ISO (1.5, 3, 4.5%), SP600125, LY2157299, ISO (1.5%) + SP600125, ISO + LY2157299, DMSO, DMSO + ISO.	Inhalation of different concentration of isoflurane (ISO) (1.5%, 3% and 4.5%) for 60 mins, starting after reperfusion. Intracerebral injection of LY2157299 or SP600125 30 minutes prior to tMCAO.	Isoflurane postconditioning decreased neurological deficits and infarct volume compared to control group, correlated with increased TGF-β1 and Smad2 expression. TGF-β1 inhibitor treatment (LY2157299) abrogated these effects.	TGF-β1 protein by IHC and WB in hippocampus. pSmad2/3 and Smad2/3 protein by WB in hippocampus.	Correlated with reduction in neurological deficits and infarct volume.	Protective.
(Q. Zhang *et al.*, 2019) [106]	Male SD rats, permanent MCAO.	20 animals per group; MCAO, BMSC, TSP4-BMSC, sham.	BMSC or TSP4BMSC suspension (1ml, 2x10^6 cells / ml), IV, 3 hours post-MCAO.	TSP4-BMSC injection increased neurological function scores compared to MCAO control and BMSC control groups.<br>*In vitro* studies showed TSP4-BMSCs upregulated TGFβ-Smad2/3 pathway.	TGF-β1 not measured.	Upregulation of TGF-β1 by TSP4-over expressing BMSCs improved neurological function.	Protective.

(Jin *et al.*, 2019) [107]	Male C57BL/6 mice, one-hour tMCAO.	3 animals per group.	Overexpression of LRG1 by injection of AAV-LRG1 into ipsilateral striatum and cortex 2 weeks prior to tMCAO.	Overexpression of LRG1 resulted in increased infarct volume and neurological deficit score, correlated with increased caspase 3 expression.<br>Increase in phosphorylation of pSMad1/5 with AAV-LRG1, but not pSmad2.<br>Increase in expression of ALK1 with AAV-LRG1. No change in expression of PAI-1 (target of Smad 2/3), suggesting action of AAV-LRG1 was primarily to upregulate ALK1, promoting TGF-β1-Smad1/5 pathways.	TGF-β1, ALK5, ALK1, TGFβR2, p-Smad1/5, Smad1, p-Smad2, Smad2 protein by WB in the brain, TGF-β1 mRNA by qPCR in brain.	Upregulated by overexpression of LRG1, promoting apoptosis and increasing infarct size and neurological deficit post tMCAO.	Harmful.
(Yeo *et al.*, 2019) [108]	Rhesus monkeys, 82–162-minute tMCAO.	2 animals per group; age matched healthy controls, acute tMCAO, sub-acute tMCAO, chronic tMCAO.	No intervention	Infarct volume and neurological score improved over time, correlated with increased CD68+ microglia/macrophages, which colocalised with TGF-β1. Suggests TGF-β1 secretion may exert anti-inflammatory effects after ischemic stroke.	TGF-β1 protein by IHC in core, peri-infarct and contralateral.	TGF-β1 localised with CD68+ microglia/macrophages in the sub-acute phase (indicating healing response).	Protective.
(Kaiser *et al.*, 2020) [109]	Female C57BL/6J mice, cortical photo thrombosis.	6 animals sham, 3 animals at 2 and 4 animals DPI, 4 at 7 DPI and 5 at 28 DPI.	No intervention.	TGF-β1 is upregulated in the ischemic stroke denervated cervical spinal cord (DPI 4, 7, 28).	TGF-β1 mRNA by ISH in the spinal cord	TGF-β1 upregulated post ischemic stroke.	Unclear.
(Chen *et al.*, 2020) [110]	Male transgenic mice (Rosa26mTmG/+ crossed with dh5-cre /ERT2-IRES-tdTomato), one-hour tMCAO.	Number of animals not disclosed.	Tamoxifen (4 mg / 25g body weight), IP, 3x dose course every 2nd day for 5 days. Diluted in corn oil.	TGFBRI expression was significantly increased post-MCAO (as was vimentin, αSMA, fibronectin). Overexpressing Let-7i in endothelial cells led to decreased TGFβRI, vascular fibrosis and infarct volume.<br>Using an antagonist of TGFBRI decreased let-7i expression,	TGF-β1R1 mRNA by qPCR and protein by IHC and WB in lesion in the brain.	Increased TGFBRI increased vascular fibrosis post-MCAO.	Harmful.

				suggesting let-7 improves post stroke injury via TGFBRI.			
(Xu *et al.*, 2020) [111]	Male C57BL/6 mice, six-hour tMCAO.	10 animals per group; sham, tMCAO, tMCAO + Danhong injection (DHI), tMCAO + BuChang NaoXinTong (BNC), tMCAO + Ginaton	MCAO performed after 5 days of each drug:<br>BNC (440 mg/kg/dose PO).<br>DHI 5 ml/kg/dose, IP injection.<br>Ginaton, 5 ml/kg/dose, IP injection. All delivered twice a day for 5 days	Smad3 and 4 were identified as critical transcription factors upregulated post-MCAO.<br>DHI and BNC both reduced infarct volume and improved NSS compared to MCAO control, correlated with decreased Smad3/4 expression.	Smad4 mRNA by qPCR in brain.	Decreased expression of Smad2/3 pathway correlated with improved outcome post-pMCAO.	Harmful.
(K. Zhang *et al.*, 2019) [112]	Male SD rats, two-hour tMCAO.	Number of animals not disclosed.	Injection of lentiviral vector into ischemic core to induce ALK5 overexpression (LV-ALK5), 2.46x10^{8} TU/ml) or LV-con-RNAi as control.	ALK5 protein upregulated at 24 h and 14 days post-MCAO compared to sham.<br>siRNA-ALK5 caused significant reduction in ALK5 protein expression, correlated with increased neurological deficit score and adhesive removal scores. Effect reversed by ALK5 overexpression.<br>pSmad2/3 downregulated with ALK5 siRNA treatment, opposite noted with overexpression.	ALK5 protein by WB and IHC in ischemic hemisphere.<br>pSmad2/3 and Smad2/3 protein by WB.	Overexpression of ALK5 correlated with improved outcomes.	Protective.
(Yang *et al.*, 2021) [113]	Male C57BL/6 and B3galt2 knockout mice, 90-minute tMCAO.	Total of 295 animals used.	Lentiviral vectors of B3galt2 ICV (lateral ventricle), 7 µl, 7 days before MCAO.<br>rTGF-β1, 1 ng/gram of bodyweight in 1.5 µl, 1 day prior to MCAO.	B3galt upregulated at protein level following MCAO.<br>Overexpression of B3galt2 with LV vector caused sig reduction in infarct volume and neurological deficit scoring at 24, 72h and 14 days post-MCAO compared to vector control. BBB compromise also reduced at 24 h post-MCAO.<br>B3galt knockdown significant reduced TGF-β1, TGFBRII and	TGF-β1, TGFβR2 and pSmad2/3 protein by IHC and WB in brain.	Treatment with TGF-β1 improved neurological outcomes post-MCAO.	Protective.

				pSmad2/3 expression at 24 and 72 h post-MCAO, with worsened neurological severity and BBB leakage. Addition of TGF-β1 abrogated this effect.			
(Zhang *et al.*, 2022) [114]	Male SD rats, 90-minute tMCAO.	24 animals per group; sham, tMCAO, tMCAO + DMSO, tMCAO + 1,25-D3, tMCAO + 1,25-D3 + vitamin D receptor agonist P5P.	1,25-D3, IV, 5 mg/kg 30 mins before reperfusion. P5P 0.4 mg/kg IV.	1,25-D3 decreased infarct volume and improved CBF at 72h post stroke, correlated with increased expression (IF) of TGF-β1, pSmad2/3 and VEGF.	TGFβ, Smad2/3, p-Smad2/3 protein by WB in ischemic penumbra cortex. TGF-β1,2 and 3 mRNA by qPCR in penumbra cortex.	TGF-β1 upregulation by 1,25-D3 produced a neuroprotective effect.	Protective.
(Rajput *et al.*, 2021) [115]	SD rats (sex not disclosed), one-hour tMCAO .	Total number of animals not disclosed.	Pretreatment with efonidipine (1 mg/kg/day) for 4 weeks prior to MCAO	Efonidipine treatment in normoglycaemic rats caused a marked reduction in TGF-β1 mRNA, correlated with reduction in both NSS and brain infarct area.	TGF-β1 mRNA by RT-PCR in brain.	Reduction in TGF-β1 correlated with improved outcomes.	Harmful.
(Y. Zhang *et al.*, 2021) [116]	Male C57BL/6 mice, one-hour tMCAO.	8 animals per group with 5 groups for part 1 of study. 6-8 animals per group for part two.	R,S ketamine – 15 mg/kg IP. Exenin 50 µg/kg, daily for 14 days. rhANP 1 mg/kg, IV, daily. NPR-A agonist A71915, 0.5 µg/kg . All drugs given daily for 14 days, starting at 14 DPI.	Ketamine improved post-stroke isolation / depressive behaviours, correlated with increased TGF-β1 expression in the CSF and improved neurological severity scoring.	TGF-β1 protein by WB in choroid plexus and ischemic hemisphere.	Increased expression correlated with improved outcomes post tMCAO, at 28 DPI.	Protective.
(Du *et al.*, 2021) [15]	C57BL/6J mice (sex not disclosed), one-hour tMCAO.	Four groups; sham, tMCAO + vehicle, tMCAO + Coicis semen (CS) (50, 100, 150 mg/kg).<br>180 total animals used.	CS prepared in 100 mM DMSO, delivered PO for 28 days prior to MCAO, and then post-MCAO for 7 and 14 days.	CS reduced BBB injury and increased VEGF expression, correlated with TGF-β1/Smad upregulation in MCAO + CS compared to MCAO + vehicle at 7 and 14 days post-MCAO. TGF-β1/Smad pathway upregulated	Smad1/5 protein by IHC in the brain. TGF-β1, ALK-1, p-Smad1/5, Smad1/5 protein	TGF-β1 increased by CS, correlating with reduced BBB injury, increased angiogenesis, reduced mortality and reduced infarct size.	Protective.

				following MCAO compared to Sham.	by WB in the brain.		
(Guo *et al.*, 2022) [117]	Male Kunming mice, one-hour tMCAO.	N=5 animals per study, but unclear how many studies were performed on same animals.	TGF-β1-siRNA, ICV injection 24 hours before tMCAO (2 µl, 300 pmol/µl)	Inhibiting TGF-β1 with siRNAs removed the protective effects (oxidative stress markers, infarct size, NSS) of rh-b3galt2 following tMCAO, with B3galt2 having previously been shown to function by activation of TGF-β1. Suggests neuroprotective effects of B3galt2 were TGF-β1 dependent.	TGF-β1 protein by WB in the brain.	Activation of TGF-β1 by B3galt2 shown to be neuroprotective, TGF-β1 silencing abrogated this effect.	Protective.
(X. Li *et al.*, 2022) [118]	Male SD rats, two-hour tMCAO.	3-4 animals per group for western blotting, immunofluorescence staining and BRD4 quantification. 4 groups of sham, control, ADV-control and ADV-shBRD4.	Adenovirus knockdown of BRD4 (ADV-shBRD4), 5 µl $1x10^9$ pfu/ml, ICV into lateral ventricle, 4 days prior to tMCAO.	tMCAO significantly upregulates BRD4 expression in rats. Knockdown of BRD4 in vivo significantly reduces fibronectin, COL1A1 and αSMA protein expression, reduces infarct volume and improves neurological function post-tMCAO. BRD4 knockdown also reduces phosphorylation of Smad2/3.	p-Smad2/3 and Smad2 protein by WB in the ischemic core.	Inhibition of BRD4 (modifying TGF-β1 signaling and reducing Smad2/3 phosphorylation) reduced infarct volume and markers of fibrosis in vivo.	Harmful.
(Shi *et al.*, 2023) [119]	Male C57BL/6 mice, 45-minute tMCAO.	6 animals per group; sham, tMCAO, tMCAO + hypoxic postconditioning (HPC)	Hypoxic postconditioning (chamber pO_2 reduced to 5%) for 45 minutes immediately after ischemia.	TGFBRI protein expression increased significantly at 14 d post-MCAO, further increase in MCAO+HPC. Correlated with spontaneous recovery in NSS and with significantly better NSS in MCAO+HPC group than MCAO control. Similar trend with beam balance test.	TGF-β1, ALK5 and p-ERK1/2 protein by WB in ischemic hemisphere.	Increased ALK5 expression correlates with improved outcomes (NSS and beam balance).	Protective.
(Hu *et al.*, 2023) [120]	C57BL/6 mice (sex not disclosed), 90-minute tMCAO .	Total number of animals not disclosed.	Nicotine 4.5 mg/kg, dissolved in saline and 5 ml/kg injected twice a day for two weeks.<br>mIR-21 in vivo	Nicotine downregulated Pdlim5 in ischemic hemisphere following MCAO. Brain tissues harvested 45 mins after MCAO showed reduction in TGF-β1 expression in ischemic hemisphere compared to non-ischemic, which was restored	TGF-β1, pSmad3 protein by IHC and WB in ischemic and non-ischemic hemisphere.	Preventing TGF-β1 depletion after MCAO linked with reduced BBB leakage.	Protective.

			transfection by ICV injection. AAV-Pdlim5 injected into striatum.	in nicotine pre-treated animals. Effect correlated with pSmad expression. Knockout of Pdlim5 ameliorated these effects, preventing TGF-β1 depletion immediately after MCAO, correlating with reduction in BBB leakage.			
(Mostajeran *et al.*, 2022) [121]	Female Wistar rats, two-hour tMCAO.	Total of 10 animals.	No intervention.	Gradual but significant recovery of neurological deficit over 14 days. Significant increase in TGF-β1 expression between ischemic core and contralateral side. Significant increase in TGFβ expression in the MCA smooth muscle layer.	TGF-β1 protein by IHC and WB in contra, peri-infarct and ischemic core.	Significantly upregulated in ischemic core and MCA smooth muscle compared to non-MCAO controls.	Protective.
(Yip *et al.*, 2012) [122]	Male SD rats, one hour tMCAO	Total of 68 animals; 12 for common physiological parameters; 8 uninjured control; 12 animals per group for control, non-acupoint, GB20 and ST36.	Electro-acupuncture preconditioning at GB20 and ST36 acupoints, either 3 treatments for 1 week prior to tMCAO or 18 treatments over 6 weeks.	Increased TGF-β1 expression in both treatment timepoints at ST36, correlated with increased BCL-2 expression.	TGF-β1 measured by WB in brain lysate from ischemic hemisphere.	Significantly upregulated by ST36 electroacupuncture treatment, correlated with BCL-2 upregulation.	Protective.

Table S3. Hemorrhagic stroke study characteristics. Animal species, model, experimental groups, intervention and reported outcomes for all included studies modelling hemorrhagic strokes.

SAS = sub-arachnoid space, ICA = internal carotid artery, ICV = intracerebroventricular, EP = endovascular perforation, DPI = days post injury, IP = intraperitoneal, ICV = intracerebroventricular, aCSF = artificial cerebrospinal fluid, BWC = brain water content, ICH = intracerebral hemorrhage, IVH = intraventricular hemorrhage, SAH = sub-arachnoid hemorrhage, GMH = germinal matrix hemorrhage.

Study	Model	Experimental groups	Intervention	Outcome summary	What/how measured	Summary of role of TGF-β1	TGF-β1 role
(Zhao *et al.*, 2019) [123]	Male SD rats, EP of ICA.	8 animals per group; ICH model and ICH + resveratrol.	Resveratrol (10 mg/kg/day) for 20 days post ICH.	Resveratrol reduced cerebral water content, hippocampus cell apoptosis and cerebral infarct volume compared to control group. Resveratrol significantly downregulated TGF-β1, and regulates apoptosis via a TGF-β1 mediated pathway (ERK).	TGFβ, ERK and p-ERK protein by WB in isolated neurons. TGF-β and ERK mRNA by qPCR in isolated neurons.	TGF-β1 promotes brain injury post cerebral hemorrhage.	Harmful.
(Hoque *et al.*, 2011) [124]	Wistar rat pups, ICV injection of 80 µl high hematocrit adult rat blood.	Colchicine group; 22 animals control, 24 treated with 20 µg/kg/day, 23 with 50 µg/kg/day. Decorin group; 23 control, 47 treated. 36 naïve control and 24 naïve animals for toxicity studies.	Colchicine at 20 or 50 µg/kg/day, split into two doses per day, delivered by gavage for 13 days starting at PN8. Decorin at 4 mg/kg/day, ICV injection on PN8 (same side as blood), followed by second dose on PN13 into opposite ventricle.	IVH model significantly worsened neuromotor outcomes and caused marked ventricular dilatation. No significant effect from decorin or colchicine.	TGF-β1 not measured.	No effect on TGF-β1 seen in this study.	Unclear.
(Kanaji, Tada and Kobayashi, 1997) [125]	C57BL/6 mouse pups, intrathecal injection of 30 µl serum, plasma or	N = 4 – 14 per group, varying between groups.	Intrathecal injection of anti-TGF-β1 antibody (30 µl of 50 µg/ml), on opposite	Serum containing TGF-β1 generated significant hydrocephalus, as did injection of hrTGF-β1 (6 ng, but not 0.6 ng).	TGF-β1 protein by WB in serum/plasma.	Induced hydrocephalus, both as injected hrTGF-β1 and as TGF-β1 in serum.	Harmful.

	TGF-β1 with leakage into SAS.		side of head as serum injection.	Marked dilation of ventricles at 12 weeks post injury.<br>Injection of anti-TGF-β1 antibody significantly reduced hydrocephalus at 6 weeks post injury.			
(Fujii *et al.*, 2014) [126]	Male SD rats, EP of MCA	20 animals sham, 31 SAH + vehicle, 33 SAH + JWH133, 19 SAH + SR144528.	All IP, 1 hr post SAH. IP injection of vehicle (0.2 ml ethanol + 1.8 ml 0..9% saline), JWH133 1 mg/kg or SR144528 3 mg/kg (15 mins prior to JWH133).	CB2R agonist significantly increased TGF-β1 expression, correlating with increased NSS, reduced BWC and increased ZO-1 protein expression compared to vehicle control.	TGF-β1 protein by WB in the ipsilateral brain.	Significantly increased with CB2R agonist treatment.	Protective.
(Wu *et al.*, 2020) [127]	Male C57BL/6 or db/db mice, collagenase injection (0.075 U) into basal ganglia.	8 animals per group; C57BL/6J treated and control, db/db treated and control.	15mg/kg IP adiponectin peptide 12 and 24 hours prior to ICH induction. 10 ng TGF-β1 in 1 µl PBS at same coordinates as ICH, 10 mins prior to ICH.	Adiponectin treatment reduced brain oedema and improved neurological function post-ICH in both normal and diabetic mice. Treatment alleviated mitochondrial dysfunction and apoptosis in a Smad3 dependent manner. Reducing pSmad3 improved the outcome of ICH.	p-Smad2/3 and Smad2/3 protein by IHC and WB in brain.	TGFβeta 1 administration abolished the protective effects of adiponectin.	Harmful.
(Yan *et al.*, 2016) [54]	Male SD rats, autologous blood intracisternal injection.	24 animals sham, 36 SAH, 18 SAH + decorin, 18 SAH + vehicle.	Two injections of rhDecorin; 30 µg/ml into cisterna magna prior to model induction, followed by ICV injection 10 days post-SAH.	TGF-β1 in CSF increased at 3,6,10,14 and 21 DPI compared to sham, with same result in brain parenchyma at all but 3 DPI. Decorin injection reduced latency testing and swim distance, indicating improved neurological outcomes, in addition to decreasing ventricle size. Decorin reduced TGF-β1 , pSmad2/3, CTGF and COl1 protein expression relative to vehicle control.	TGF-β1 protein by ELISA in CSF and IHC and WB in brain. P-Smad2/3, pERK1/2 protein by WB in brain.	Decorin reduced TGF-β1 / pSmad2/3 and CTGF, improving neurological outcomes and preventing ventricular enlargement from SAH.	Harmful.
(Ayer *et al.*, 2013) [128]	Male SD rats, endovascular	24 animals sham, 32 SAH + vehicle, 33 SAH + 1 mg/kg simvastatin,	1.5 ml IP injection of 1 and 20 mg/kg of simvastatin 30	Animals treated with high dose simvastatin showed improved NSS and higher levels of TGF-β1 in the	TGF-β1 protein by WB and IHC measured in	Protective against SAH.	Protective.

	perforation of ICA.	26 SAH + 20 mg/kg simvastatin.	minutes after induction of SAH. Vehicle 1.5 ml of 10% ethanol in saline.	cortex and brainstem compared to SAH. T cells in the subarachnoid space were increased following SAH and simvastatin.	cortex and brainstem in each group.		
(Dong *et al.*, 2018) [55]	Male SD rats, autologous blood intracisternal injection.	25 animals per group; sham, SAH, SAH + ICAII (1 mg/kg), SAH+ICAII (5 mg/kg), SAH+ICAII (10mg/kg)	Daily oral gavage of ICAII (1, 5 or 10 mg/kg), starting 24 h post-SAH induction, for 21 days.	Expression of TGF-β1, p-Smad2/3 and CTGF upregulated in tissue following SAH, with increase in neurological severity. Hydrocephalus incidence (+lateral ventricle size) greater in SAH group, reduced with ICAII. TGF-β1 protein levels in CSF, and TGF-β1 + CTGF RNA in tissue also decrease with ICAII dosage.	TGF-β1 protein by ELISA in CSF, TGF-β1 and CTGF mRNA by qPCR in brain and TGF-β1, p-Smad2/3, Smad2/3 and CTGF protein by WB in brain.	Reduction in TGF-β1 expression in tissue and CSF correlates with reduced hydrocephalus and reduced CTGF expression.	Harmful.
(Taylor *et al.*, 2017) [129]	Male mice, transgenic B6.SJL-Ptprca Pep3b/BoyJ (CD45.1), C57/BL6J (WT), and B6.129P-Cx3cr1tm1Litt/J (Cx3cr1GFP/GFP and Cx3cr1+/GFP, injected with whole blood or collagenase (0.05 U).	N = 7-12 per group, varying throughout.	10 ng TGF-β1 or vehicle in 1 µl. Pretreatment with injection into right striatum 10 mins prior to ICH. Post-treat by ICV injection 4 h post ICH.	Treatment with TGF-β1 improves functional outcomes post ICH. ELISA on brain homogenate post-ICH showed significant decrease in IL6 and increase in TGF-β1 at 14DPI.	TGF-β1 protein by ELISA in perihematomal region. P-Smad2 protein by IHC in brain.	Suggested to reduce pro inflammatory cytokine production.	Protective.
(Liao *et al.*, 2016) [56]	Male SD rats, intracisternal injection of autologous blood (0.5 ml).	34 animals sham, 28 SAH control, 29 SAH + LSKL – N15, 29 SAH + LSKL.	1 mg/kg LSKL, IP, following model induction and every 12 hours until sacrifice.	LSKL suppresses SAS fibrosis by inhibiting TSP1 and TGF-β1 with reduced pSmad2/3 expression. Correlated with improved neurological outcomes (Morris water maze), and lateral ventricle index, indicating reduced hydrocephalus.	TGF-β1 and thrombospondin-1 by ELISA in CSF. pSMad2/3 and Smad2/3 protein by WB.	Reduction in TGF-β1 correlated with improved outcomes and reduced hydrocephalus following SAH.	Harmful.

(Regnier-Golanov *et al.*, 2021) [130]	Male C57BL/6J mice, EP of circle of Willis.	3 animals sham, 4 SAH, 3 naïve.	No intervention.	Increases in TGF-β1 and TGFβr2/3 expression in hippocampal tissue post SAH.	TGF-β1 mRNA by next-generation sequencing.	Increased post SAH.	Harmful.
(Cherian *et al.*, 2004) [131]	Wistar rat pups, ICV injection of rat blood or artificial CSF (aCSF).	5 animals sham, 26 no surgery control, 36 blood injected, 30 aCSF injected.	No intervention.	aCSF and blood injection both generated ventricular dilation compared to control. Animals with hydrocephalus showed increase in TGF-β1 staining in ependymal cells. Peri-vascular fibronectin and laminin consistently higher in hydrocephalus.<br>Double staining with TGF-β1 and GFAP confirmed TGF-β1 localization around blood vessels in hydrocephalic brains.	TGF-β1, 2 and 3 and p44/42 MAP kinase protein by IHC in the brain.	TGF-β1 expression seems to correlate with development of post-hemorrhage hydrocephalus.	Harmful.
(Wen *et al.*, 2023) [132]	Male C57BL/6 mice, collagenase (0.3 U) into basal ganglia.	4-15 animals per group; sham + vehicle, sham + TGF-β1, ICH + vehicle, ICH + TGF-β1.	TGF-β1 IP (2 µg per mouse) or normal saline, 1 h post ICH.	Significant increase in survival at 7 days for mice injected with TGF-β1 compared to control, with reduction in hematoma volume at DPI3 and 7. Significant increase in NSS. Reduction in BBB leakage and TNFα expression in TGF-β1 group.	TGF-β1 not measured.	Improves survival, reduces BBB damage and exerts anti-inflammatory effects.	Protective.
(Finkel *et al.*, 2023) [58]	New Zealand white rabbit pups, glycerol ICV injection.	5 pups per group; No IVH, IVH + saline, IVH + USSC	ICV injection of human cord blood (hCB)-derived unrestricted somatic stem cells (USSCs). $1x10^6$ cells in 10 µl normal saline per ventricle.	Reduction in TGF-β1 mRNA (tissue) and protein (CSF) following intraventricular hemorrhage, rescued back to control levels with USSC injection. Gradual decline in TGF-β1 in control from PND1-7-14.	TGF-β1 protein by ELISA in the CSF and TGF-β1 mRNA by qPCR in brain.	Reduced following IVH and rescued with USSCs, associated with proliferation of cells.	Protective.
(Carmichael *et al.*, 2008) [133]	Male C57BL/6 mice, injection of 10 µl autologous blood (2.5 mm deep to surface).	For RT-qPCR studies, 6 animals sham, 6 ICH and 7 control. For immunostaining assays, 6 control and 6 ICH.	No intervention.	TGF-β1 not induced in mouse ICH (RT-qPCR) but is in humans.	TGF-β1, TGFβ2, TGFβ3, TGFβR2 mRNA by qPCR .in	TGF-β1 does not contribute to mouse ICH severity.	Unclear.

					perihematomal tissue.		
(Aquilina *et al.*, 2008) [134]	Wistar rat pups, ICV injection of 80 µl high hematocrit blood.	43 animals total; 21 control (water injected) and 22 pirfenidone treated.	300 mg/kg/day pirfenidone or 40 / 200 mg/kg/day losartan given by gavage twice a day PN8-PN21.	Pirfenidone and losartan had no effect on ventricular volume or neuromotor tests following IVH.	TGF-β1 not measured.	Oral anti TGFβ drugs had no effect on IVH.	Unclear.
(Manaenko *et al.*, 2014) [135]	SD rat pups, collagenase (0.3 U) ICV injection.	92 animals total; 20 animals sham, 44 GMH vehicle control, 28 GMH + SD208.	SD208 (TGFBRI inhibitor) in DMSO, 20 / 60mg/kg, IP injection. Either injected daily for 3 days, starting 1 h post GMH, or daily for 3 days starting 3 DPI.	TGF-β1 level increased at 3 and 6 h post GMH induction.<br>SD208 reduced pSMAD2/3 after GMH compared to vehicle control Acute treatment with SD208 (60mg/kg) reduced developmental delay (righting reflex) at 1 and 2 DPI, and NSS at 24 DPI. Also reduced brain tissue volume loss and ventricular dilation.	TGF-β1 and TGFβR1 protein by WB.	Inhibiting TGF receptor 1 reduced pSMAD2/3, development delay, NSS, brain atrophy and ventricular dilation when used at 60 mg/kg compared to control.	Harmful.
(Tan *et al.*, 2017) [57]	Male SD rats, ICV injection of 200 µl autologous blood.	23 animals per group; sham, vehicle, JWH-133, JWH-133+SR144528.	CBR2 agonist JWH-133 (injected at 1.5 mg/kg IP 1 and 24 h post-model induction. CB2 antagonist SR144528 injected at 3mg/kg 5 mins prior to JWH injection.	CB2 agonists decreased ventricle size and escape latency, while improving forelimb placing score and crossing times at 14 DPI compared to vehicle control MCAO, with effect abrogated by CB2 antagonist.<br>Decrease in SAS and ventricular wall fibrosis with CB2 agonist, effect removed with CB2 antagonist, assessed by staining and western blot.<br>TGF-β1 mRNA in tissue and protein in CSF followed the same effect.	TGF-β1 mRNA by qPCR in brain tissue and TGF-β1 protein by ELISA in CSF.	Reduced TGF-β1 expression at 14 DPI correlated with reduced SAS / ventricular wall fibrosis and improved neurological outcomes.	Harmful.
(Yang *et al.*, 2019) [136]	Male piglets, right frontal injection of autologous blood (1 ml followed by	Part 1 – 12 animals total; 4 per group culled at days 1 and 3 post injury, plus 4 sham. Part	Minocycline, IM, 4mg/kg 2 hours after ICH, then 2 mg/kg	MRI assessment revealed reduced brain swelling with minocycline treatment, with reduced neurological deficit compared to	TGFβ and TGFβR2 mRNA by qPCR in brain. TGFβ	Minocycline may exert a protective effect by upregulating TGF-β1.	Protective.

	1.5 ml 5 minutes later).	2 – 28 animals total; three and 14 days after surgery for RT-PCR (n = 4 per group, day 3), immunohistochemistry (n = 3 per group per day), Western blot assay (n = 4 per group, day 3), and electron microscopy (n = 3 per group, day 14).	every 12 hours for 3 days.	control group. TGF-β1 mRNA upregulated, with down-regulation of iNOS, TNFα and IL1β.	protein by IHC and WB in white matter.		
(Ren *et al.*, 2021) [137]	Male CD-1 mice, intracerebral injection of collagenase (0.075 U).	387 animals total.	Dextrose (hyperglycemic group), 3 h post ICH, 6 ml/kg of 50% dextrose IP.<br>TMF (Aryl hydrocarbon (AHR) receptor antagonist) or 3-methylcloranthere (AHR agonist) administered IP.	Hyperglycemia worsened outcomes post-ICH, with increased hematoma volume, BWC and increased NSS. Effects abrogated by AHR inhibitor. Suggests that thrombospondin-1 - TGF-β1/VEGF signaling pathway contributes to AHR signaling after ICH.	TGF-β1 and Thrombospondin-1 protein by WB in brain.	TGF-β1 pathways activation by AHR signaling may worsen outcome post ICH.	Harmful.
(Deng *et al.*, 2023) [138]	Male CD-1 mice, intrastriatal injection of autologous blood (30 µl).	138 animals sham, 242 ICH.	rCCL17 1 h post-ICH, intranasal, 30ug/kg.<br>SB431542 (TGFβ inhibitor) 1 h pre-ICH, IP, 1 µM solution, 100 µl/animal.<br>CD25-specific monoclonal antibody or isotype control antibody 48 h pre-ICH delivered ICV.	Blockage of TGF-β1 receptor activation in ICH resulted in reduced neurological outcomes, reduced TGF-β1 protein expression and reduced pSmad2/3.	TGFβ, p-Smad2/3 and smad2/3 protein by WB in ipsilateral brain tissue.	Increased TGF-β1 expression and pSmad2/3 increased neurological outcomes post ICH, abrogated by administering TGF-β1 antagonist.	Protective.

Table S4. TGF-β1 concentrations from included studies, interpreted using WebPlotDigitizer.

Reference	Method	Location	Control value	Model value	Units	Timeframe (days)
(Howe *et al.*, 2019) [67]	Luminex	Cortex	3.7	11.7	pg/mg	21
(Ma *et al.*, 2024) [76]	ELISA	Serum	457	147	pg/ml	1
(Zhu *et al.*, 2002) [91]	ELISA	Brain	23	-	pg/g	0
	ELISA	Plasma	1683	-	pg/ml	0
(Pang *et al.*, 2001) [79]	ELISA	Contralateral hemisphere	63.6	266.7	pg/g	1
	ELISA	Ipsilateral hemisphere	60.1	186.2	pg/g	1
(Cekanaviciute *et al.*, 2014) [96]	ELISA	Peri-infarct cortex	20	53.2	pg/mg	2
(Lou *et al.*, 2018) [104]	ELISA	Brain	1.5	3.4	ng/g	1
(Yan *et al.*, 2016) [54]	ELISA	CSF	6.6	75.5	pg/ml	3
				60.2	pg/ml	6
				96.3	pg/ml	10
				92.2	pg/ml	14
				82.9	pg/ml	21
(Dong *et al.*, 2018) [55]	ELISA	CSF	27	84.9	pg/ml	21
(Taylor *et al.*, 2017) [129]	ELISA	Peri hematoma region	261	28.5	pg/ml	1
				46.3	pg/ml	3
				143.4	pg/ml	7
				297.6	pg/ml	10
				343.9	pg/ml	14

(Liao *et al.*, 2016) [56]	ELISA	CSF	34.7	315.5	pg/ml	3 to 5
(Finkel *et al.*, 2023) [58]	ELISA	CSF	515.7	411.5	ng/ml	3
			536.1	362.6	ng/ml	7
			321.1	226.1	ng/ml	14
(Tan *et al.*, 2017) [57]	ELISA	CSF	9.9	69.3	pg/ml	14

Table S5. SYRCLE Risk of Bias (RoB) scoring for all included studies.

Reference	Number of SYRCLE RoB criteria met (0-10)<br>*(Low score = high risk of bias)*
**Hemorrhagic**	
(Zhao *et al.*, 2019) [123]	5
(Hoque *et al.*, 2011) [124]	8
(Kanaji, Tada and Kobayashi, 1997) [125]	2
(Fujii *et al.*, 2014) [126]	5
(Wu *et al.*, 2020) [127]	5
(Yan *et al.*, 2016) [54]	5
(Ayer *et al.*, 2013) [128]	4
(Dong *et al.*, 2018) [55]	6
(Taylor *et al.*, 2017) [129]	3
(Liao *et al.*, 2016) [56]	3
(Regnier-Golanov *et al.*, 2021) [130]	5
(Cherian *et al.*, 2004) [131]	5
(Wen *et al.*, 2023) [132]	3
(Finkel *et al.*, 2023) [58]	3
(Carmichael *et al.*, 2008) [133]	1
(Aquilina *et al.*, 2008) [134]	6
(Manaenko *et al.*, 2014) [135]	5
(Tan *et al.*, 2017) [57]	6
(Yang *et al.*, 2019) [136]	5
(Ren *et al.*, 2021) [137]	8
(Deng *et al.*, 2023) [138]	9
**Ischemic**	
(Abdel-Rahman *et al.*, 2020) [59]	7

(Li *et al.*, 2018) [60]	1
(Yang *et al.*, 2015) [61]	4
(Kong *et al.*, 2019) [62]	6
(Liu *et al.*, 2015) [63]	4
(J. Li *et al.*, 2022) [64]	4
(Dong *et al.*, 2016) [65]	4
(G. X. Zhang *et al.*, 2019) [66]	5
(Howe *et al.*, 2019) [67]	8
(Che *et al.*, 2019) [68]	4
(Yin *et al.*, 2020) [69]	4
(Jiang *et al.*, 2016) [70]	5
(Becker *et al.*, 2003) [71]	2
(Sugimoto *et al.*, 2014) [53]	3
(Gliem *et al.*, 2012) [72]	4
(Zhu *et al.*, 2017) [73]	3
(Yu *et al.*, 2018) [74]	0
(Nguyen *et al.*, 2021) [75]	3
(Ma *et al.*, 2024) [76]	2
(Lehrmann *et al.*, 1998) [77]	3
(Long *et al.*, 2023) [78]	1
(Pang *et al.*, 2001) [79]	2
(Meng *et al.*, 2016) [14]	3
(Vincze *et al.*, 2010) [80]	2
(Cai *et al.*, 2015) [81]	4
(Wang *et al.*, 1995) [82]	2
(Henrich-Noack, Prehn and Krieglstein, 1996) [83]	1
(Lin *et al.*, 2016) [84]	3
(Buscemi *et al.*, 2019) [85]	2
(Islam *et al.*, 2018) [16]	3
(Ma *et al.*, 2008) [86]	4
(Pál *et al.*, 2012) [87]	3

(Ruocco *et al.*, 1999) [88]	2
(Xin *et al.*, 2013) [89]	2
(Gross *et al.*, 1993) [90]	1
(Zhu *et al.*, 2002) [91]	3
(Huang *et al.*, 2023) [92]	8
(L. Zhang *et al.*, 2021) [93]	7
(Ali *et al.*, 2001) [94]	2
(Leung *et al.*, 2002) [95]	1
(Cekanaviciute *et al.*, 2014) [96]	4
(Liu *et al.*, 2013) [97]	4
(Yoo *et al.*, 2013) [98]	7
(Cheng *et al.*, 2015) [99]	1
(Pál, Lovas and Dobolyi, 2014) [100]	3
(Zhang *et al.*, 2014) [101]	6
(Song *et al.*, 2017) [102]	6
(Fernández-López *et al.*, 2016) [103]	0
(Lou *et al.*, 2018) [104]	5
(Wang *et al.*, 2016) [105]	3
(Q. Zhang *et al.*, 2019) [106]	4
(Jin *et al.*, 2019) [107]	2
(Yeo *et al.*, 2019) [108]	3
(Kaiser *et al.*, 2020) [109]	4
(Chen *et al.*, 2020) [110]	8
(Xu *et al.*, 2020) [111]	4
(K. Zhang *et al.*, 2019) [112]	2
(Yang *et al.*, 2021) [113]	5
(Zhang *et al.*, 2022) [114]	4
(Rajput *et al.*, 2021) [115]	2
(Y. Zhang *et al.*, 2021) [116]	7
(Du *et al.*, 2021) [15]	6
(Guo *et al.*, 2022) [117]	5

(X. Li *et al.*, 2022) [118]	3
(Shi *et al.*, 2023) [119]	5
(Hu *et al.*, 2023) [120]	3
(Mostajeran *et al.*, 2022) [121]	4
(Yip *et al.*, 2012) [122]	4

Figure S1. Fixed effects random plot of ischemic stroke studies.

Study or Subgroup	TGF-β1 Mean	TGF-β1 SD	TGF-β1 Total	Control Mean	Control SD	Control Total	Weight	Std. mean difference IV, Fixed, 95% CI
73 (5 ng TGF-β1; HI)	0.11	0.03	10	0.17	0.04	10	24.7%	-1.63 [-2.67 , -0.58]
79 (AdRSVahTGF-β1; ICV)	15.77	1.86	8	27.14	3.48	8	8.1%	-3.85 [-5.68 , -2.03]
86 (1 µg TGF-β1; IN)	41.75	4.72	4	51.91	9.67	6	13.4%	-1.12 [-2.54 , 0.29]
90 (10 µg TGF-β1; ICA)	16.7	10.6	7	31.9	17.5	7	20.9%	-0.98 [-2.12 , 0.15]
90 (50 µg TGF-β1; ICA)	21.7	11.9	7	31.9	17.5	7	22.8%	-0.64 [-1.72 , 0.45]
91 (AdRSVahTGF-β1; ICV)	15.64	2.03	10	27.07	3.31	10	10.0%	-3.99 [-5.62 , -2.35]
**Total**			**46**			**48**	**100.0%**	**-1.62 [-2.13 , -1.10]**

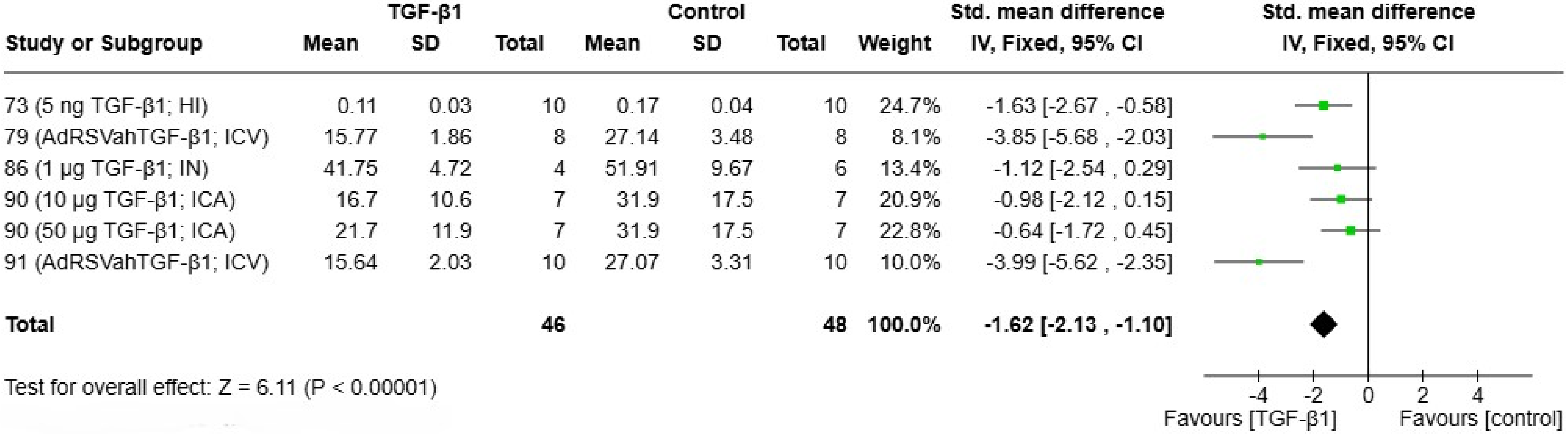

Test for overall effect: Z = 6.11 (P < 0.00001)

Heterogeneity: Chi² = 18.66, df = 5 (P = 0.002); I² = 73%

Figure S2. Fixed effects forest plot of hemorrhagic stroke studies.

Study or Subgroup	TGF-β inhibitor Mean	SD	Total	Control Mean	SD	Total	Weight	Std. mean difference IV, Fixed, 95% CI
124 (Decorin; ICV)	4.39	2.88	47	3.93	1.58	23	27.0%	0.18 [-0.32 , 0.68]
125 (TGF-β1 antibody; IT; acid serum model)	0.36	0.08	14	0.5	0.03	7	5.3%	-1.97 [-3.09 , -0.85]
125 (TGF-β1 antibody; IT; crude serum model)	0.38	0.07	19	0.51	0.07	4	4.5%	-1.79 [-3.01 , -0.57]
134 (High dose Losartan; G)	6.82	3.53	15	10.06	5.45	12	10.9%	-0.70 [-1.49 , 0.08]
134 (Low dose Losartan; G)	8.19	6.85	13	10.06	5.45	12	10.8%	-0.29 [-1.08 , 0.50]
134 (Pirfenidone; G)	6.6	4.19	22	6.48	4.34	20	18.4%	0.03 [-0.58 , 0.63]
135 (Acute SD208; IP)	27.82	3.92	8	57.86	13.23	8	2.9%	-2.91 [-4.43 , -1.39]
135 (Delayed SD208; IP)	29.62	4.8	8	57.86	13.23	8	3.2%	-2.68 [-4.13 , -1.23]
54 (Decorin; ICV)	2.79	1.19	12	4.06	1.6	12	9.5%	-0.87 [-1.71 , -0.03]
56 (LSKL; IP)	2.84	1.08	10	4.15	1.32	10	7.5%	-1.04 [-1.99 , -0.09]
**Total**			**168**			**116**	**100.0%**	**-0.57 [-0.83 , -0.31]**

Test for overall effect: Z = 4.32 (P < 0.0001)

Heterogeneity: Chi² = 41.47, df = 9 (P < 0.00001); I² = 78%

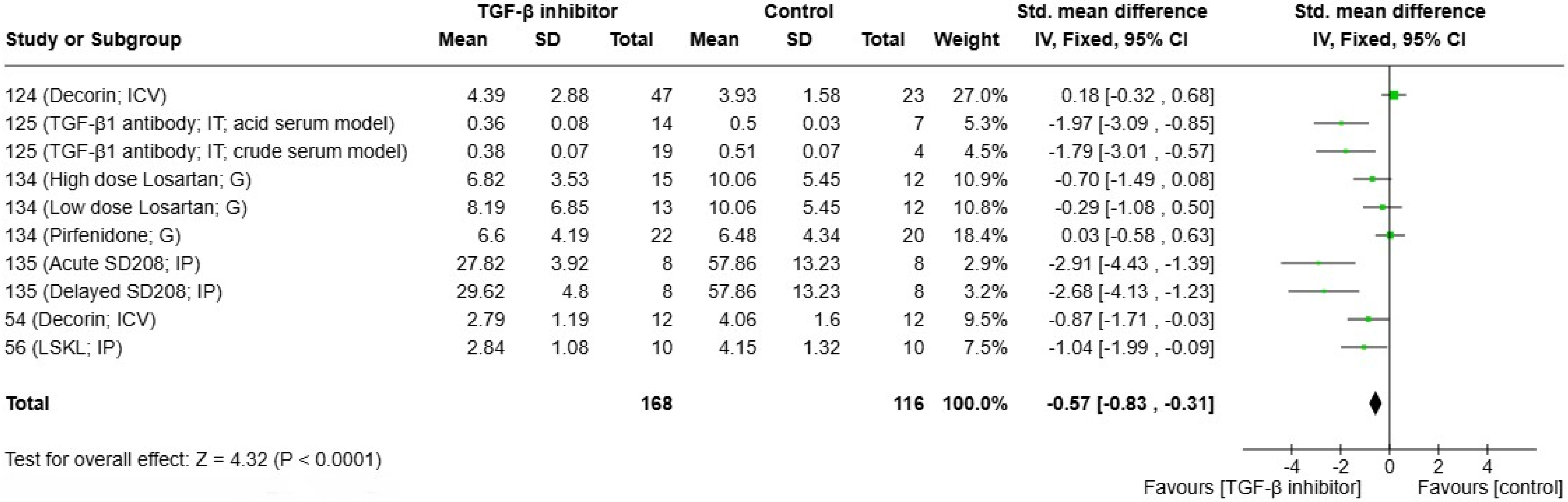

Supplement: Supplementary file 1 — Tables S1–S5 Figures S1–S2 [file JAH3-14-e037890-s001.pdf]
